# Opaque-2 induced zein reduction and lysine increase suggest a route to quality protein sweet corn

**DOI:** 10.3389/fpls.2026.1814115

**Published:** 2026-05-14

**Authors:** Jonathan Niyorukundo, Abou Yobi, Caleb Wehrbein, Ruthie Angelovici, David R. Holding

**Affiliations:** 1 Department of Agronomy and Horticulture, University of Nebraska, Lincoln, NE, United States; 2 Department of Biological Sciences, University of Missouri, Columbia, MO, United States

**Keywords:** biofortification, lysine, quality protein maize, sweet corn, zeins

## Abstract

The zeins make up over 60 percent of the kernel total proteins in maize subspecies including sweet corn. However, zeins are devoid of the essential amino acid lysine rendering the protein content of sweet corn incomplete. The *opaque-2* mutation reduces the accumulation of the zeins in maize endosperm, subsequently increasing lysine-containing non-zeins. In this study, the objective was to introduce the *opaque-2* mutation from Quality Protein Maize (QPM) varieties *(o2* donor) into *sugary-1* and *shrunken-2* sweet corn varieties to produce high-lysine sweet corn, namely quality protein sweet corn (QPS). To do this, two QPM varieties were crossed to sweet corn parents. The F_1_ plants were self-pollinated to produce F_2_ seeds which visibly segregated for *opaque-2* and sweet corn phenotypes. Sweet corn kernels were advanced through the inbred phase by selecting for the *opaque-2* mutation at kernel maturity, sweetness and flavor, and texture phenotypes at the prime eating stage (20 days after pollination; DAP). Six QPS lines were selected for *o2* uniformity, satisfactory sweetness and flavor, and tender texture. These QPS lines were then used to produce 12 F_1_ QPS hybrids. The analysis of lysine and other amino acid profiles in six QPS inbred and 12 QPS hybrid lines revealed that all inbreds and hybrids had increased protein-bound lysine, and some lines had higher free lysine compared to the corresponding wild-type sweet corn parents. Furthermore, biochemical assays revealed that QPS sugar and starch contents were similar to parental sweet corn. This study shows proof of concept for the ability of the *opaque-2* mutation to improve sweet corn protein quality without incurring penalties on its quality traits. This can ultimately contribute to enhanced nutrition and profitability of this important vegetable crop.

## Introduction

Sweet corn is a widely consumed vegetable but is less consumed in some developing countries. However, its protein content, predominantly zeins, is incomplete because of its low levels of the essential amino acid, lysine (Kodrzycki et al., 1989). Lysine is crucial for the growth and metabolic functions of the human body. Conversely, the human body cannot synthesize lysine and relies on dietary lysine-rich foods to meet lysine needs (Gunarathne et al., 2025). In maize kernels, lysine is available in free form but mostly in protein bound. Therefore, lysine is limited in maize as the lysine-devoid zein proteins comprise 60 percent of maize kernel endosperm (Li and Song, 2020).

The mutation in the *OPAQUE-2* (*O2*) gene that regulates protein storage in maize endosperm has shown potential for reducing the zeins resulting in increased non-zeins through proteome rebalancing (Parsons et al., 2020; Zhao et al., 2023). The *opaque-2 (o2)* mutant displayed increased lysine resulting from increased non-zein proteins (Mertz et al., 1964). However, *o2* rendered the mutant kernel endosperm soft and fragile (Geetha et al., 1991). These phenotypes are problematic when it comes to seed harvest, storage and transportation. Therefore, it can result in a reduction of yield due to physical damage and rot, causing an unnecessary cost to farmers. To address this issue, breeders at the International Maize and Wheat Improvement Center (CIMMYT) made introgression of the *o2* mutation into dent corn and bred for unknown *o2* modifiers into the new variety of dent corn, named Quality Maize Protein (QPM) (Villegas et al., 1992). QPM displayed optimal kernel vitreousness (hard endosperm) and maintained the high lysine content equivalent to that of the *o2* mutant. Furthermore, Ren et al. (2018) and Parsons et al. (2020) developed QPM popcorn (QPP) inbreds and hybrids. They crossed QPM to popcorn to introgress the *o2* mutation into popcorn and selected for *o2* modifiers. The resultant popcorn displayed increased lysine content and good popcorn popping and agronomic traits (Parsons et al., 2021).

There have been previous studies aimed at exploiting the introgression of *o2* mutation in sweet corn (Jompuk et al., 2020; Mehta et al., 2020; Baveja et al., 2021; Sharma et al., 2024). Jompuk et al. (2020) introgressed *opaque-2* (*o2o2*) along with purple *(Pr1Pr1C1C1)* mutations into *shrunken-2* (*sh2sh2*) sweet corn, targeting to improve the sweet corn’s anthocyanin, and tryptophan contents. Furthermore, Mehta et al. (2020) introgressed *β-carotene hydroxylase1* (*crtRB1*) and *o2* mutations in *sh2*-based sweet corn inbreds using marker-assisted backcrossing breeding to study their effects on the contents of provitamin A, lysine, and tryptophan. Again, Sharma et al. (2024) reported the development of quality protein sweet corn using marker-assisted backcross breeding. Another study by Baveja et al. (2021) crossed QPM hybrids equipped with *opaque2* (*o2*), *lycopene epsilon cyclase* (*lcyE*) and *β‐carotene hydroxylase* (*crtRB1*) mutations with *sh2* sweet corn in effort to develop biofortified sweet corn hybrids. Nonetheless, our study extends this body of work using several unique approaches. Unlike other studies, which focused primarily on introgression of the *o2* mutation often coupled with other functional traits in *shrunken2*-based sweet corn lines, we used two distinct QPM inbreds to breed *o2* allele into two genetically and physiologically distinct sweet corn backgrounds (*su1* and *sh2*). Furthermore, this study largely targeted to explore potential effects of QPM background on the sweet corn’s normal eating qualities such as kernel sweetness and texture; hence, the selection for potentially improved protein sweet corn lines prioritized acceptable kernel palatability at prime-eating stage.

Sweet corn was bred for *sugary-1* (*su1*) and/or *shrunken-2* (*sh2*) mutations that render its sweet taste and tender texture (Szymanek et al., 2015). Previous breeding efforts have introgressed the *o2* trait into the super sweet corn (*shrunken-2*) to further improve its protein quality (Mehta et al., 2020; Singh et al., 2025). Previously, the *o2* studies in dent corn and popcorn revealed that *o2* imparts collateral changes to the kernel endosperm vitreousness. Kernel vitreousness is very important in dent corn to prevent physical damage during harvest, pests and rot during storage. In popcorn, kernel vitreousness is essential for popping quality. However, for sweet corn, kernel vitreousness is not an issue because the prime-eating stage ranges between 20 and 25 days after pollination (DAP) after which sugar starts converting into starch (Tracy, 1993). Furthermore, the soft starchy endosperm associated with the *o2* accumulates the most as the kernel matures such that the impact on the sweetness might be minimal. Singh et al. (2025) showed that *o2* did not reduce the sweetness in *o2* biofortified super sweet corn. The majority of the *o2* sweet corn biofortification has prioritized the *shrunken-2* varieties owing to its high sweetness and popularity in the current market. Traditional *sugary-1* sweet corn varieties have not been a target for *o2* introgressions.

Over the past 40 years, *sh2*-based sweet corn made up the majority of commercial sweet corn on the market (Hu et al., 2021; Branch et al., 2024). Today, traditional *sugary-1(su1)* sweet corn varieties comprise only 30% of the commercial sweet corn and consumers prefer the *sh2* varieties because of the higher sugar contents and longer shelf life after harvest (Dodson-Swenson and Tracy, 2015). However, *su1* sweet corn have stronger germination and seedling vigor than *sh2* sweet corn. Also, *su1* sweet corn has a unique flavor and texture still appreciated by consumers (Lago et al., 2014). That said, in this study, our objective was to introgress the *opaque-2* allele in mainly *sugary-1* sweet corn and one *shrunken-2*-based sweet corn variety as the control. We used a conventional breeding approach to breed the high lysine trait into various publicly available sweet corn inbred lines to improve their overall protein content. We further sought to observe any difference in the response to the *o2* introgression among the sweet corn with the *su1* allele and *sh2* alleles. Two QPM lines were crossed to eight sweet corn inbreds, followed by successive self-pollinations for five generations. At each generation, QPS lines were selected for the *o2* mutation and sweet corn kernel phenotypes such as sweetness and tender texture. Inbreds were then crossed to make F_1_ hybrids and preliminary testing of all the hybrids was performed using a single location. The resultant selected QPS lines displayed increased fresh kernel lysine content relative to their parental sweet corn lines. Moreover, the sugar and starch content of QPS did not change substantially compared to the sweet corn parents. Breeding *o2* into sweet corn increased the lysine thus resulting in an enhanced protein profile. The best hybrids identified from this preliminary study will next be used for multilocation field trials.

## Materials and methods

### Germplasm, site location, and field design

Seeds were obtained from the public seed stock centers and multiple small seed suppliers (Mary’s Heirloom seeds, Sherwood’s seeds, and Baker Creek Heirloom seeds). Eight sweet corn varieties and two QPM parent varieties were acquired. The sweet corn parents (S1-S8) were IA5125, IA453, P51, P39, NE-EDR *su1*, NE-EDR *sh2*, FL2, and FL56 respectively and the QPM parents (QPM1 and QPM2) were CML154 and TX807 respectively. The planting site was located at the University of Nebraska-Lincoln East Campus field, Lincoln, NE (40.83486°N, 96.66377°W). The field was rain-irrigated in the Summer of 2020, 2021, and 2022. Drip-irrigation was used in the summer of 2023 and 2024. In 2020 and 2021, seeds were planted in a single row per genotype and were not replicated for early selection. From 2023 genotypes were planted in a Completely Randomized Design (CRD). Genotypes were planted in a block of six rows, each 3.65 m long and 76.2 cm apart. A 60 cm border alley separated two adjacent blocks.

### Hybridization, selection, and production of QPS inbreds and hybrids

**Figure 1:**
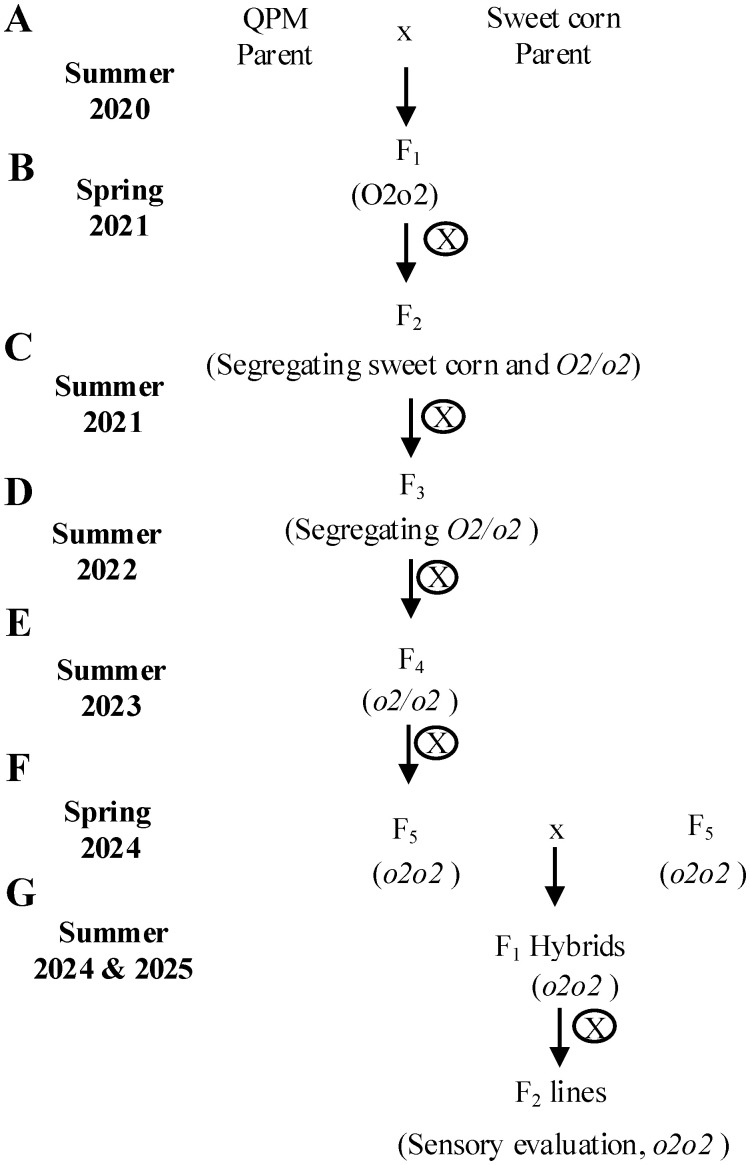
Breeding scheme for producing QPS F_1_ hybrids. **(A)** in the summer of 2020, QPM parents were crossed with sweet corn parents bidirectionally to create F_1_ QPS heterozygotes. **(B)** in the spring of 2021, heterozygotes were self-pollinated to generate segregating F_2_ QPS offsprings. Sweet corn kernels were selected based on their shrunken phenotype and no selection of *o2* mutations were conducted. **(C)** in the summer of 2021, F_2_ sweet corn were self-pollinated to generate F_3_ offspring, homozygous for sweet corn phenotype and segregating for *o2* mutation. Kernels showing opaque phenotype were selected. **(D)** in the summer of 2022, F_3_ *o2* heterozygotes and homozygotes were self-pollinated to generate F_4_ QPS offsprings. Whole ears showing full opaque phenotype were selected. **(E)** in the summer of 2023, F_4_ homozygotes were again self-pollinated to generate F_5_ fully homozygous QPS inbreds. **(F)** in the spring of 2024, high performing QPS inbreds were outcrossed to produce F_1_ QPS hybrids. **(G)** in the summer of 2024, F1 QPS hybrids were self-pollinated to generate F_2_ lines to evaluate their sensory performance.

The acquired seed parents were bulked first, and different combinations of crosses were made, followed by the successive selections of sweet corn lines at each generation with successful *o2* introgression, desirable sugar and starch content, and good plant and seed agronomics as described (**Figure 1**). In the summer of 2020, initial crosses were made among QPM and wild-type sweet corn varieties to produce new *o2* sweet corn mutants. In the spring of 2021, the F_1_ heterozygotes seeds were self-pollinated in the greenhouse to produce segregating F_2_ lines. The F_2_ ears segregated for dent corn and sweet corn phenotypes; *o2* heterozygotes and homozygotes. Mature F_2_ kernels with shrunken sweet corn phenotype were selected, bulked and, in the summer of 2021, they were self-pollinated in the field to produce F_3_ *o2* heterozygote sweet corn lines. Opaque phenotypes were visually verified on the light box (**Supplementary Figure 1**) and were confirmed using SDS PAGE. Selected o*2* seeds were bulked and were self-pollinated in the field for two successive generations (summer of 2022 and 2023) to produce homozygous *o2* F_5_ sweet corn lines, QPS inbreds (**Table 1**). In the spring of 2024, F_1_ hybrids (**Table 2**) were made from a combination of outcrosses between best performing QPS inbreds in the greenhouse. Using a single location for preliminary tests to identify the best lines which would later be used for multiple location testing, F_1_ QPS hybrids were grown and were self-pollinated to produce QPS hybrid-derived F_2_ lines in the summer of 2024 and 2025.

**Table 1:** Pedigree details for the development of QPS F_5_ inbreds.

Ref. No.	Pedigree
QPS1	(IA5125su1/CML154) → F_1B_ → F_2B_ → F_3B_ → F_4B_ → F_5_
QPS2	(IA453su1/CML154) → F_1B_ → F_2B_ → F_3B_ → F_4B_ → F_5_
QPS3	(CML154/P51su1) → F_1B_ → F_2B_ → F_3B_ → F_4B_ → F_5_
QPS5	(NE-EDRsu1/CML154) → F_1B_ → F_2B_ → F_3B_ → F_4B_ → F_5_
QPS6	(NE-EDRsh2/CML154) → F_1B_ → F_2B_ → F_3B_ → F_4B_ → F_5_
QPS7	(P39su1/TX807) → F_1B_ → F_2B_ → F_3B_ → F_4B_ → F_5_

Six final QPS inbreds were generated. Inbreds were selected for o2 trait and sweet corn eating qualities. “B” stands for bulking. For simplicity, reference numbers QPS1-QPS7 were used for identification of inbreds in this analysis. Sweet corn parents were referenced as S1: IA5125su1, S2:IA453su1, S3:P39su1, S4:P51su1, S5: NE-EDRsu1, S6:NE-EDRsh2.

**Table 2:** Pedigree details for the development of QPS experimental F_1_ hybrids.

Ref. No.	Crosses	Pedigree
H1	QPS1+QPS2	(IA5125/CML154) F_5_ + (IA453/CML154) F_5_
H2	QPS1+QPS3	(IA5125/CML154) F_5_ + (CML154/P51) F_5_
H3	QPS1+QPS5	(IA5125/CML154) F_5_ + (NE-EDRsu1/CML154) F_5_
H4	QPS1+QPS6	(IA5125/CML154) F_5_ + (NE-EDRsh2/CML154) F_5_
H5	QPS2+QPS3	(IA453/CML154) F_5_ + (CML154/P51) F_5_
H6	QPS2+QPS5	(IA453/CML154) F_5_ + (NE-EDRsu1/CML154) F_5_
H7	QPS2+QPS7	(IA453/CML154) F_5_ + (P39/Tx807) F_5_
H8	QPS3+QPS5	(CML154/P51) F_5_ + (NE-EDRsu1/CML154) F_5_
H9	QPS3+QPS6	(CML154/P51) F_5_ + (NE-EDRsh2/CML154) F_5_
H10	QPS3+QPS7	(CML154/P51) F_5_ + (P39/Tx807) F_5_
H11	QPS5+QPS6	(NE-EDRsu1/CML154) F_5_ + (NE-EDRsh2/CML154) F_5_
H12	QPS6+QPS7	(NE-EDRsh2/CML154) F_5_ + (P39/Tx807) F_5_

12 total QPS hybrids were developed in the winter of 2024. For simplicity, reference numbers H1-H12 were used for identification of hybrids in this analysis.

### Harvest, processing, and storage of eating stage sweetcorn

Fresh sweet corn ears were harvested at 20 DAP. Four to six biological replicates of each genotype were hand-picked and transported in a cooler to the laboratory for long-term storage. Before freezing, replicates were husked, arranged with a label showing their genotype and pollination date, digitally imaged, and plunge frozen in liquid nitrogen. Each frozen ear was transferred to individual labelled resealable bags and larger bags for organization and then stored at -80° Celsius.

### Protein extraction and SDS-PAGE zein profiling

Zein proteins were extracted and profiled to confirm the presence of *o2* mutation in the QPS lines. The confirmation procedures were done using SDS PAGE on extracted zein and non-zein proteins as described and modified from Wallace et al. (1990). Briefly, 3–4 fresh (20 DAP) seeds were picked from the frozen QPS lines at -80 degrees Celsius. Then, seeds were placed in small coin envelopes and were lyophilized for 24–36 hours. For protein extraction, one frozen seed was ground into a fine flour in liquid nitrogen using a pistil and a mortar. To extract seed protein from mature dry QPS lines, a single dry seed was ground with a Heavy-duty Wig-L-Bug^®^ grinder/mixer analog (Sigma-Aldrich, Catalog no. Z111392) for 1 minute. 50 mg samples of flour were weighed and placed into a 2.0 ml microcentrifuge. 1.6 ml of Borate extraction buffer were added followed with sample homogenization using a vortex mixer. After, the samples were placed on Orbit™ LS Low Speed Laboratory Shaker and were incubated at room temperature for 2 hours. After, samples were centrifuged 13,300 g for 15 minutes to fractionate the total proteins from non-proteins (lipids and carbohydrates). After centrifugation, the supernatant containing total proteins were transferred to a new 2 ml microcentrifuge tube. To fractionate the zein proteins from other proteins, non-zein proteins were precipitated out by adding 300 ml of total protein extract to 700 ml of 100% ethanol. Then, the samples were mixed and were incubated overnight at 4 degrees Celsius. After, the samples were centrifuged at 13,300g for 15 minutes. Zein supernatants were dry evaporated in a vacuum concentrator. The zein pellets were resuspended in 40 ml of 1x SDS loading buffer. The non-zein pellets were washed in 70% ethanol twice to remove residual zeins and were partially air-dried and resuspended in 300 ml of deionized water. The samples were stored at 4 degrees Celsius for immediate use or -20 degree Celsius for long term storage. After zein sample preparation, SDS PAGE were performed to separate different bands of zein proteins to confirm the presence of *o2* mutation.

### Determination of sugar and starch contents of QPS inbreds and hybrids

Sucrose and total starch analyses were performed to determine the sugar contents in QPS inbreds and hybrids. Sucrose and total starch contents were determined using the Megazyme Sucrose/D-Glucose Assay Protocol (CAT NO. K-SUCGL) and Total Starch Assay Protocol (REF K-TSTA-100A; 700004351) respectively. Both sucrose and total starch assays measure the absorbance values of quinoneimine dye that is produced from the oxidation of D-Glucose by glucose oxidase/peroxidase (GOPOD) enzyme. The amount of quinoneimine dye produced is stoichiometrically equal to the amount of D-Glucose. To measure sucrose and total starch from the sample, D-Glucose was extracted first. Briefly, eight to ten seeds were picked from the frozen biological replicates and lyophilized according to the aforementioned procedures above for flour preparation. To measure the sucrose content, 50 mg of sample were diluted with 100 ml of deionized water to make a sample extract at a concentration of 0.5 mg/ml. Then, 0.2 ml of sample extract were poured into four 16 x 10 mm glass test tubes. Then, 0.2 ml of acetate buffer (prepared according to the assay protocol) and 0.2 ml of b-fructosidase were added to duplicate tubes. After, tubes including the reagent blanks and D-Glucose controls were incubated at 50 degrees Celsius for 20 minutes. 3.0 ml of GOPOD reagent were added to all tubes and incubated again at 50 degrees Celsius for 20 minutes. Then, absorbances were measured at 510 nm against the reagent blank using a MegaQuant™ Wave Spectrophotometer. To measure the total starch content, 100 mg of sample were washed with 80% v/v aqueous ethanol and dimethyl sulphoxide to remove free D-Glucose or maltodextrins and resistant starch respectively. Then, diluted thermostable a-amylase were added to extract D-Glucose which were oxidized with GOPOD reagent to measure the absorbance value of quinoneimine dye produced. The absorbance values were used to calculate the total starch content as described in the Megazyme assay protocol.

### Analysis of amino acid profile in QPS inbreds and hybrids

The amino acid profiles of QPS lines were analyzed to determine the effects of *o2* mutation in QPS lines compared to the wild-type sweet corn varieties. A pool of 8–10 seeds were taken from the frozen ears and were lyophilized and prepared as described in the aforementioned procedures above. Protein-bound amino acids (PBAA) and free amino acids (FAA) in the QPS inbreds and hybrids were extracted and analyzed as described (Ansaf et al., 2025a, b). Briefly, FAA were extracted from 4 mg sample using 13 internal standards and the sample were analyzed using ultra-performance liquid chromatography-tandem mass spectrometer (UPLC-MS/MS) instrument (Waters Corporation, Milford, MA) (Angelovici et al., 2013). For PBAA extraction, 3 mg flour were hydrolyzed (Fountoulakis and Lahm, 1998) with 6N HCl for 24 hours at 110°C. 10 µl hydrolysate were dried and the pellets were resuspended in FAA extraction buffer (13 internal standards). Solutions were processed and analyzed using the FAA analysis protocol. Data were processed using the MassLynx data analysis software (TargetLynx XS, Waters, Inc.).

### Preliminary sensory evaluation of sweetness and texture of QPS inbreds and hybrids

Sweetness and texture of QPS inbreds and hybrids were evaluated at 20 days after pollination (DAP) to assess sweetness and texture phenotypes in the new *o2* sweet corn varieties. Three to five fresh QPS ears were harvested and were immediately tasted raw by at least three testers. At least three separate ears were tested to account for plant to plant variation. Then, a consensus score for taste (1 being bland, 5 being sweet) and texture (1 being tough, 5 being tender) were assigned. Furthermore, plants were visually evaluated for their physical phenotypes such as height, pollen quality, ear size and weight, and early/late pollination period. The qualities were used in selection of superior QPS lines at each generation and later used in hybrid production.

### Statistical analysis

Data were presented as Means ± SEM. A one-way analysis of variance (ANOVA) was performed followed by Tukey’s Honestly Significant Difference (HSD) multiple comparison test to determine statistical differences and significance between colored popcorn parents and colored QPP inbreds. Significance was shown as asterisks: *p < 0.05; **p < 0.01; ***p < 0.001, and ns: not significant.

## Results

### Confirmation of *opaque-2* trait and phenotypes in QPS inbreds and hybrids

**Figure 2:**
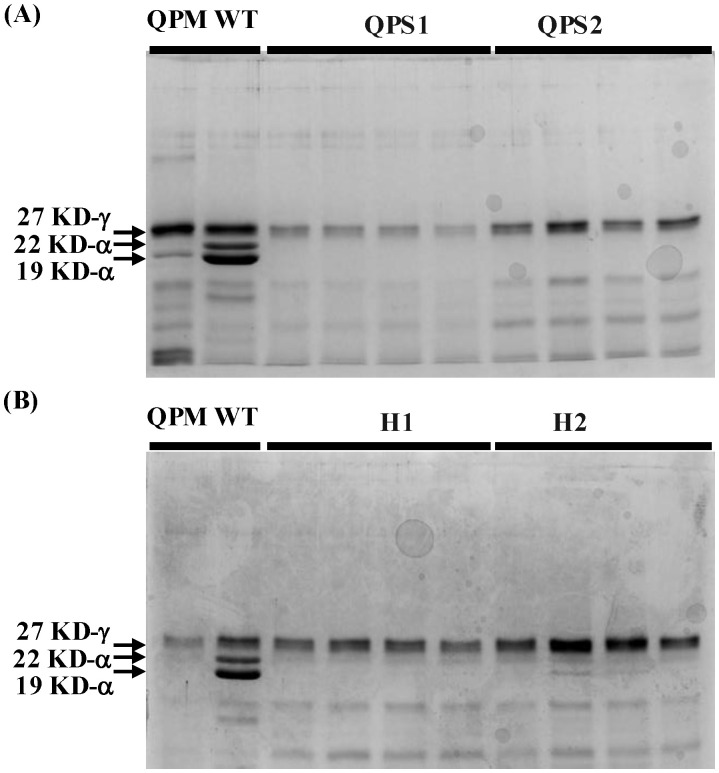
SDS-PAGE confirmation of *opaque-2* introgression in QPS inbreds and hybrids. Freshly harvested QPS ears (20 DAP) were used for protein extraction. Zein protein analysis of QPS inbreds and hybrids showed a significant reduction of the 19-kDa and 22-kDa α-zein proteins consistent with the QPM zein profile. **(A)** SDS-PAGE gel of the zein proteins of QPM, wild type sweet corn, and F_5_ QPS inbreds. **(B)** SDS-PAGE gel of the zein proteins of QPM, wild type sweet corn, and F_2_ lines derived from F_1_ QPS hybrids.

To confirm successful introgression of *o2* into wild-type sweet corn, the zein fraction of QPS inbreds and hybrids were extracted and analyzed using SDS PAGE. The analysis of the zein profiles showed a substantial difference between the QPM-derived sweet corn (QPS inbreds and hybrids) and the wild-type sweet corn (**Figure 2**; **Supplementary Figures 2**, **S3**). Even at 20 DAP, wild-type sweet corn exhibited the strong accumulation of both the 19-kDa α−zein and the 22-kDa α-zeins, similar to mature seed zein composition. However, the zein profile of the QPS inbreds (**Figure 2A**; **Supplementary Figure 3**) and the QPS hybrids (**Figure 2B**; **Supplementary Figure 4**) showed a strong reduction of the 19-kDa α-zein and very low levels of the 22-kDa α-zein, which is consistent with the zein profile of dent corn-based QPM. QPM lines exhibit a strong knockdown of the 22-kDa α−zein but maintain low 19-kDa α-zein. Overall, the shift in the zein profile of the QPS lines to match the QPM zein profile confirms a successful *o2* introgression into the wild-type sweet corn highlighting the potential of a conventional breeding approach to improve protein quality in sweet corn.

### Total protein concentration in QPS inbreds and hybrids

**Figure 3:**
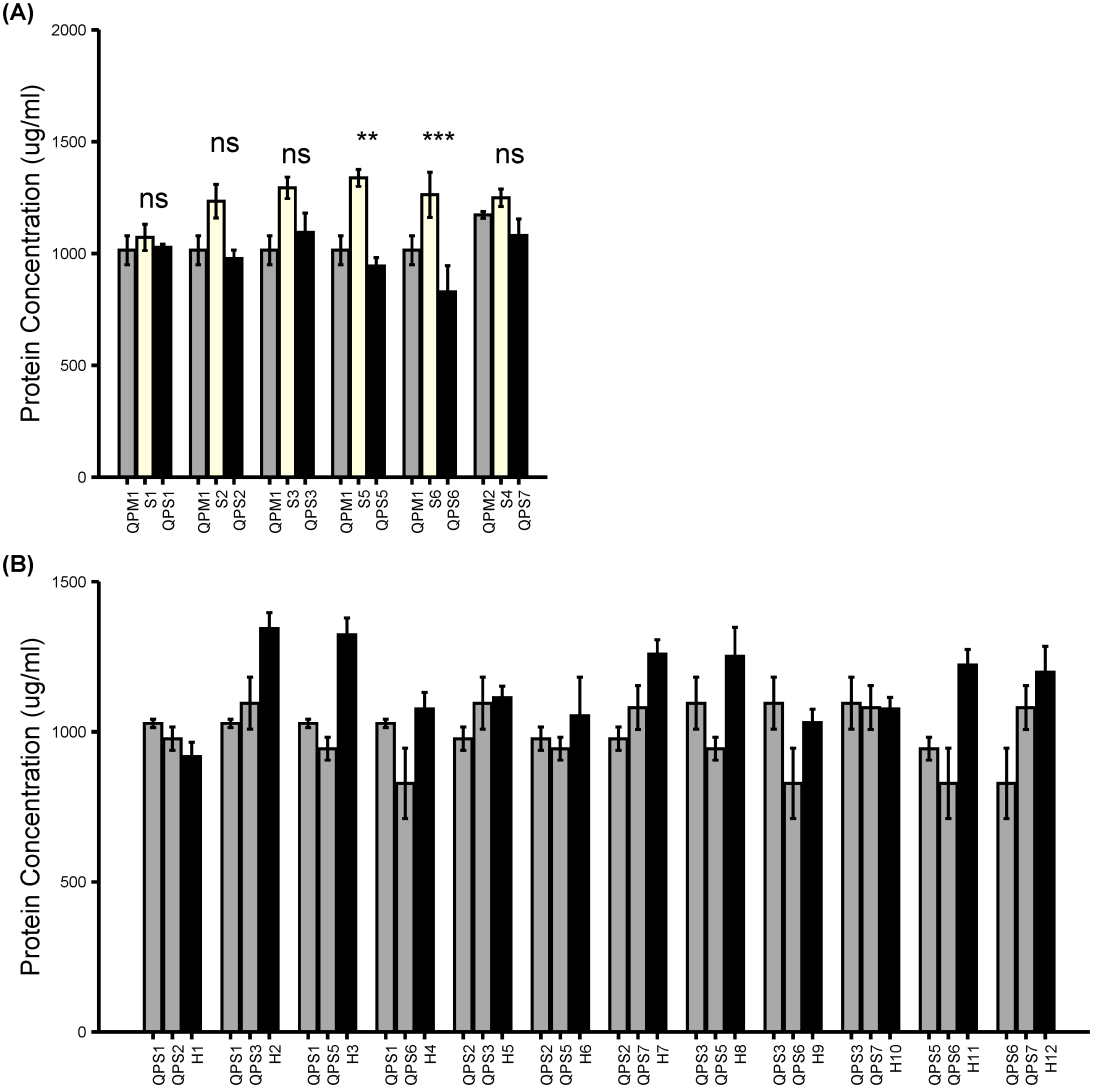
BCA Total protein analysis of *opaque-2* introgression in QPS inbreds and hybrids. Freshly harvested QPS ears (20 DAP) were used for protein extraction. **(A)** Total protein concentrations in the QPM, parental sweet corn, and QPS inbreds. **(B)** Total protein concentrations in the parental QPS inbreds and QPS hybrids. Results are shown as mean ± SE (n = 4). Statistical difference between parental sweet corn, colored sweet corn inbreds and hybrids was determined using one-way ANOVA followed by Tukey’s HSD test. The asterisks show significant differences: **p < 0.01; ***p < 0.001, and ns, not significant.

The relative comparison of the total protein concentration among the developing QPS inbreds and the parental sweet corn displayed slightly varying and unexpected outcomes in some QPS inbreds. QPS inbreds lines exhibited protein concentrations nearly equivalent to the QPM and normal sweet corn parents except QPS5 and QPS6 which had significantly lower protein concentrations compared to their respective parental sweet corn (**Figure 3A**). In fact, the reduction in the protein concentration noted in QPS5 and QPS6 inbreds were very significant (p< 0.01). The varying protein concentrations in the QPS inbreds depended on the individual sweet corn parent. For instance, the protein concentration in QPS1 was 4.11% lower than the S1; QPS2 were 18.76% lower than the S2; QPS3 was 15.37% lower than S3; QPS5 29.43% lower than S5; QPS6 was 40.92% lower than S6; QPS7 were 13.5% lower than S4. This observation indicates that the parental sweet corns responded differently on the *o2* introgression. Despite the consistent reduction of the protein concentrations in the QPS inbreds, nine of the 12 QPS hybrids had higher protein concentrations than both of the parental QPS inbreds (**Figure 3B**). The protein increase varied among the hybrids with some hybrids including H2, H3, H7, H11, and H12 showing significant increase compared to one or two of the parental inbreds. It appears that the heterosis effect in the hybrid kernels resulted in increased total protein.

### QPS inbreds and hybrids have increased lysine compared to wild type sweet corn

**Figure 4:**
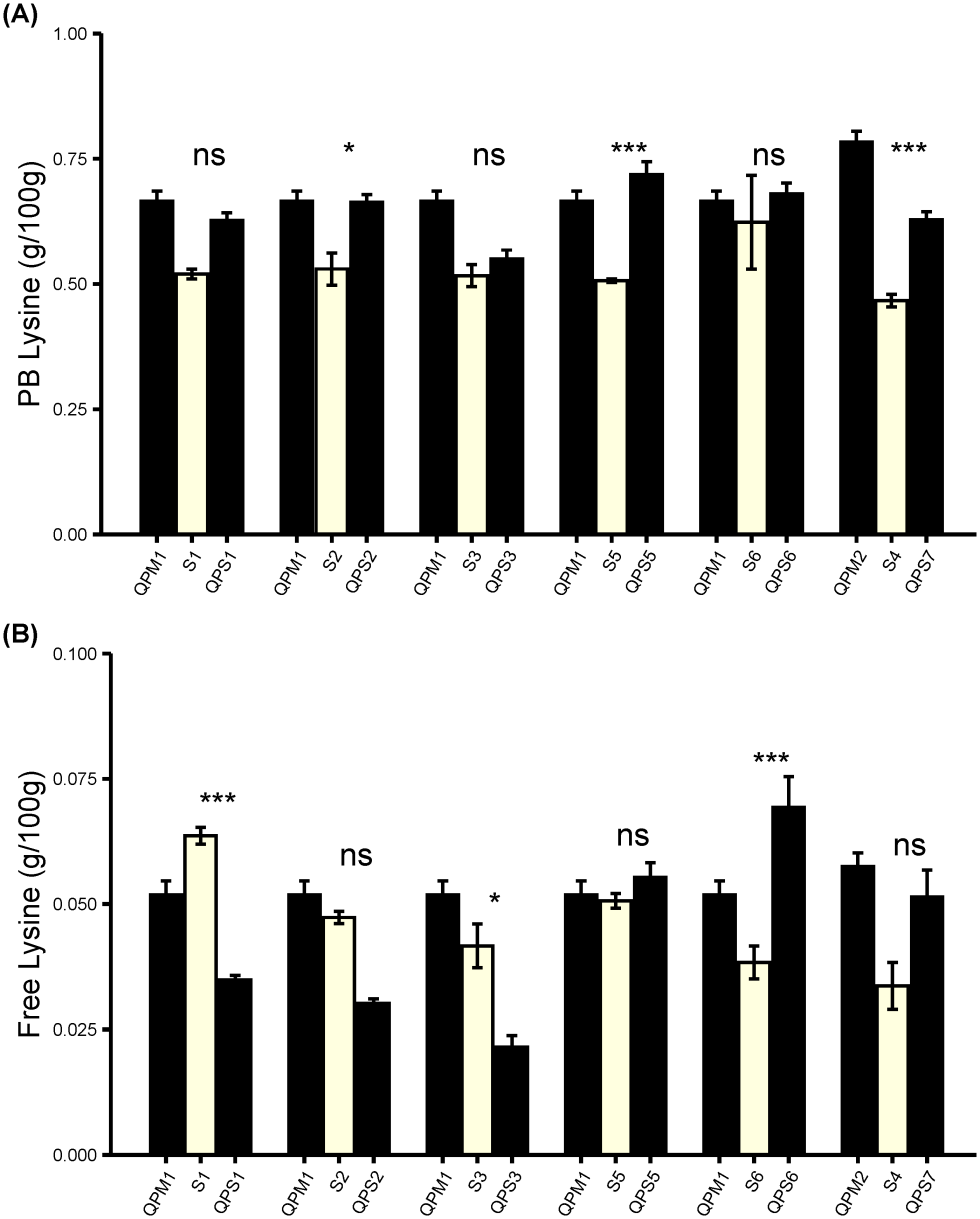
Profiling amino acids in six F_5_ QPS. Fresh ears harvested at 20 DAP were used for amino acid extraction. **(A)** Protein-bound lysine. **(B)** Free lysine in F_5_ QPS inbreds. Results are shown as mean ± SE (n = 6). Statistical difference between parental sweet corn, colored sweet corn inbreds and hybrids was determined using one-way ANOVA followed by Tukey’s HSD test. The asterisks show significant differences: *p < 0.05; ***p < 0.001, and ns, not significant.

**Figure 5:**
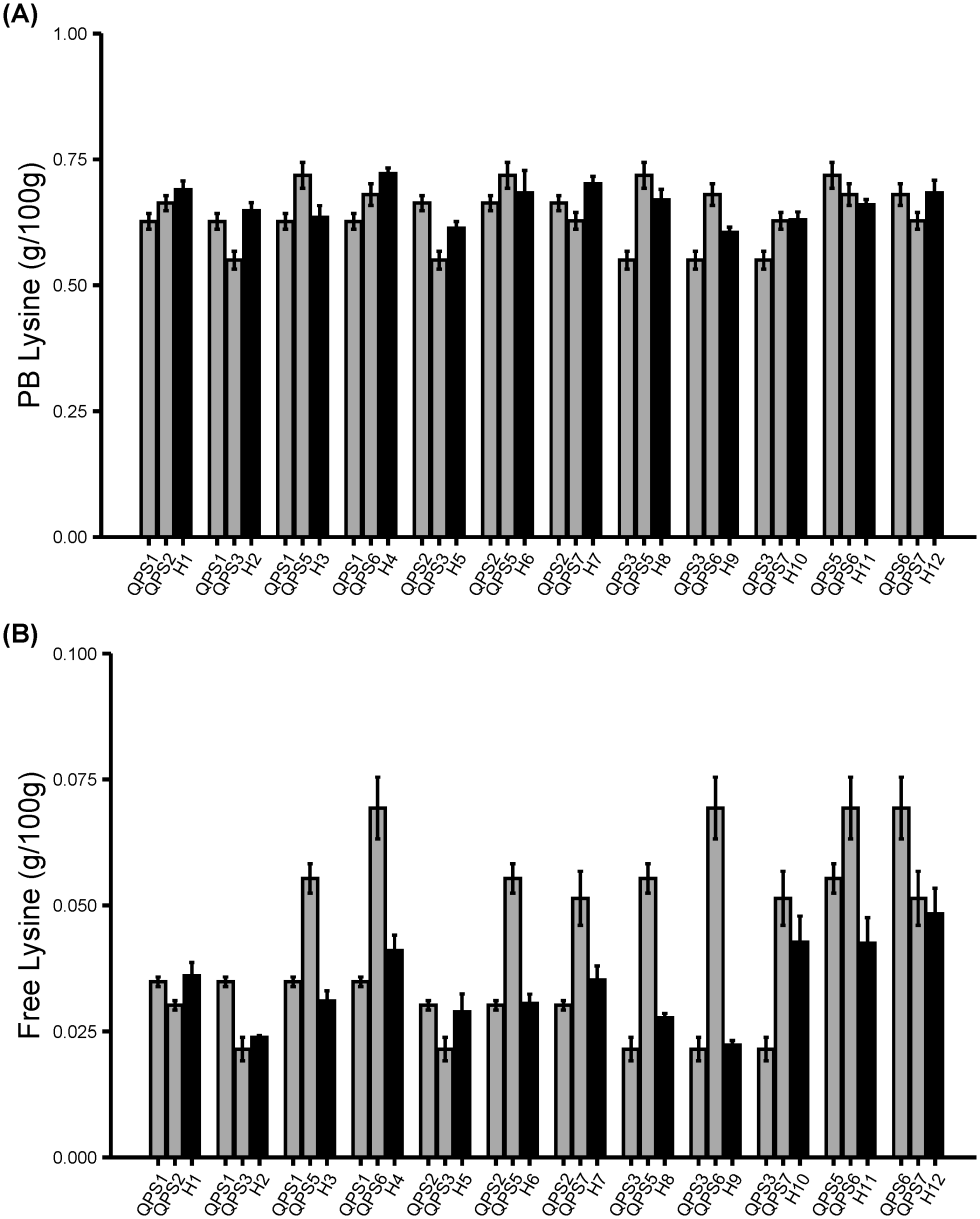
Profiling amino acids in 12 F_2_ lines derived from F_1_ QPS hybrids. Fresh ears harvested at 20 DAP were used for amino acid extraction. **(A)** Protein-bound lysine. **(B)** Free lysine. Results are shown as mean ± SE (n = 6).

To confirm the *opaque-2* mutation and resulting zein reductions increased the lysine content in the QPS inbred and hybrid lines, Protein-bound Amino Acid (PBAA) and Free Amino Acid (FAA) assays were performed (**Figure 4**). The *o2* introgression in sweet corn resulted in increased PB lysine in all the six QPS inbreds lines and the PB lysine content in QPS inbreds were comparable to those observed in the QPM lines (**Figure 4A**). The PB lysine increase was significant in four of the six QPS lines and the percentage PB lysine difference between the QPS and WT increase ranged between 6.5% and 41.8% (**Supplementary Table 1**). In contrast, the free lysine was only increased in three QPS inbreds lines and was significantly lower in the other three QPS inbreds lines compared to the WT (**Figure 4B**; **Supplementary Table 2**). However, the reduction of the free lysine is expected to have a very small effect since the free lysine content were at least 8X lower than the PB lysine content (**Supplementary Table 3**). Furthermore, the lysine contents in QPS hybrids were compared against the lysine content in the parental QPS inbreds. QPS hybrid lines displayed the PB lysine (**Figure 5A**) and free lysine (**Figure 5B**) contents consistent with that of the inbreds from which they were made. Overall, the *o2* increased PB lysine and free lysine as high as 1.55- and 1.8-fold with some lines exhibiting PB lysine and free lysine 1.06 and 0.52 fold compared to the WT (**Supplementary Table 5**). These results emphasize varying response of the sweet corn lines to the *o2* introgression. Nonetheless, the *o2* gene shows the potential to improve the lysine content in sweet corn thereby enhancing its overall protein quality.

### QPS inbreds and hybrids displayed varying effects on other amino acids profiles

The method used to extract and profile amino acids does not allow protein bound tryptophan to be quantified which requires a separate protocol. However, it is well established that an approximate 4:1 ratio of lysine to tryptophan occurs in maize kernels which is maintained across variants (Hernandez and Bates, 1969). As a result, protein-bound lysine or tryptophan concentrations are often used as proxies for one another. It is therefore likely that the protein-bound tryptophan increased given that the protein-bound lysine was increased across the QPS lines (**Figures 4A**, **5A**). In contrast, the results did not show substantial effects on the free tryptophan concentrations across the QPS inbreds except QPS1 which had almost double the concentration compared to the respective parental sweet corn (**Supplementary Figure 11**, **Supplementary Table 2**). Although free tryptophan was measured, free amino acids contribute much less to the overall amino acids content since their concentrations are approximately 1/10 that of protein bound. Overall, because of the lack of protein-bound data and the low concentrations of free tryptophan, we did not make direct conclusions about tryptophan.

The *opaque-2* gene contributed to the changes in lysine contents of the QPS inbred and hybrid lines. Besides lysine, the analysis of other essential and conditionally essential protein-bound and free amino acids was performed to assess further *opaque-2* implications on their profiles (**Supplementary Figures 4**–**S12**). Results indicated that the protein-bound contents of isoleucine (**Supplementary Figure 4**), leucine (**Supplementary Figure 5**), and phenylalanine (**Supplementary Figure 6**) decreased in all the six QPS inbreds lines; two QPS inbreds and five QPS inbreds lines showed increased contents of the protein-bound methionine (**Supplementary Figure 7**) and threonine (**Supplementary Figure 8**) respectively. Moreover, the protein-bound contents of histidine and valine remained relatively unchanged across all six QPS inbred lines and other conditionally essential amino acids, with the exception of proline, were increased in the majority of the QPS lines (**Supplementary Figures 9**, **S10**). In contrast, results also indicated that nearly all QPS inbred lines showed increased free amino acid contents compared to the parental sweet corn (**Supplementary Figures 11**, **S12**).

The *opaque-2* mutation strongly affected the protein-bound isoleucine content. The protein-bound fraction decreased significantly in all six QPS lines compared to the parental QPM and sweet corn lines (**Supplementary Figure 4A**). In contrast, the free isoleucine increased in all six QPS lines, with some of the QPS inbreds showing even higher free isoleucine than the QPM lines (**Supplementary Figure 4B**). The PB isoleucine in QPS inbreds lines decreased 1.48 to 1.88 fold whereas the free isoleucine increased 1.15 to 177 fold compared to the WT (**Supplementary Table 5**). In contrast, the QPS hybrids had protein-bound isoleucine content higher than the corresponding parental QPS inbreds (**Supplementary Figure 4C**). Though the PB isoleucine was lower in the QPS inbreds relative to the parental sweet corn, the PB isoleucine was restored in all the 12 QPS hybrids. Consistently, the QPS hybrids had free isoleucine content equivalent or higher to one or both parental QPS inbreds (**Supplementary Figure 4D**). Though initially the *opaque-2* mutation lowered PB isoleucine in the parental QPS inbreds, some levels of PB isoleucine were recovered in the QPS hybrids and the free isoleucine content.

Leucine, the isomer of isoleucine, was also strongly affected by the *opaque-2*. The PB leucine decreased nearly by half in all the six QPS inbred lines (**Supplementary Figure 5A**). The percentage decrease ranged from 1.74 to 2.22 fold compared to the WT (**Supplementary Table 5**). The reduction of PB leucine was significant in all six QPS inbreds (P<0.05). In contrast, similar to the isoleucine, the free leucine increased in all the QPS inbred lines compared to the parental sweet corn, and a few QPS inbred lines showed higher free leucine content than the QPM lines (**Supplementary Figure 5B**). Furthermore, protein-bound leucine (**Supplementary Figure 5C**) and free leucine (**Supplementary Figure 5D**) in the hybrids remained consistent relative to the parental inbreds. Generally, the protein-bound leucine fraction reacted negatively to *opaque-2* while the free leucine fraction reacted positively (**Supplementary Table 3**).

Phenylalanine was also among the essential amino acids that were affected strongly by the *opaque-2* introgression. The protein-bound fraction of phenylalanine decreased significantly in all six QPS inbred lines relative to the parental sweet corn (**Supplementary Figure 6A**). The PB phenylalanine reduction the in QPS inbred lines ranged between 1.46 and 176 fold while the increase of free phenylalanine in same QPS lines ranged from 1.1 to 2.41 fold (**Supplementary Table 5**). The increase in free phenylalanine was significant in QPS1, QPS5, and QPS7. However, the concentration of the free Phe was at least 10X lower than the PB Phe (**Supplementary Table 3**). Furthermore, the results showed that all QPS hybrids had consistently higher PB phenylalanine than the parental QPS inbreds (**Supplementary Figure 6C**). The free phenylalanine content in QPS hybrids were equivalent or higher than one of the parental QPS inbreds (**Supplementary Figure 6D**). Similar to leucine and isoleucine, the phenylalanine protein-bound fraction was persistently lower in the QPS inbreds than the parental sweet corn, but they slightly increased in the QPS hybrid lines, while free phenylalanine was increased.

The effects of the *opaque-2* mutation on methionine were not as consistent as they were for isoleucine, leucine, and phenylalanine. Only QPS1 and QPS2 inbred lines had increased PB methionine contents while four QPS inbreds had reduced PB methionine compared to their parental sweet corn (**Supplementary Figure 7A**). Additionally, free methionine increased significantly in QPS1, QPS2, and QPS7 (**Supplementary Figure 7B**). The PB methionine contents in the QPS hybrids did not change from the parental inbred lines (**Supplementary Figure 7C**). However, the free methionine contents in QPS hybrids varied greatly across the hybrids. (**Supplementary Figure 7D**). Overall, the *opaque-2* showed a more positive impact on the free methionine than the protein-bound methionine in the QPS lines.

Similarly to lysine, threonine reacted positively to the introgression of *o2* in sweet corn. There were higher amounts of PB threonine in five of the six QPS inbred lines compared to the parental sweet corn (**Supplementary Figure 8A**). PB threonine increased significantly in QPS1 and QPS7 by 1.13 and 1.24 fold (**Supplementary Table 5**) while other QPS inbreds remained relatively the same as the WT. Consistently, free threonine was higher in all six QPS inbreds compared to the parental sweet corn and QPM lines with some QPS lines increasing by 3 and 6 times. (**Supplementary Figure 8B**). Importantly, the results indicated that the protein-bound threonine contents were consistently higher in all QPS hybrids than the parental inbreds (**Supplementary Figure 8C**). The free threonine content indicated a varying degree of response in the QPS hybrids (**Supplementary Figure 8D**). Overall, *o2* did not have adverse effects on the threonine amino acid compared to other affected essential amino acids.

### QPS inbreds and hybrids had similar sugar and starch content to sweet corn parents

**Figure 6:**
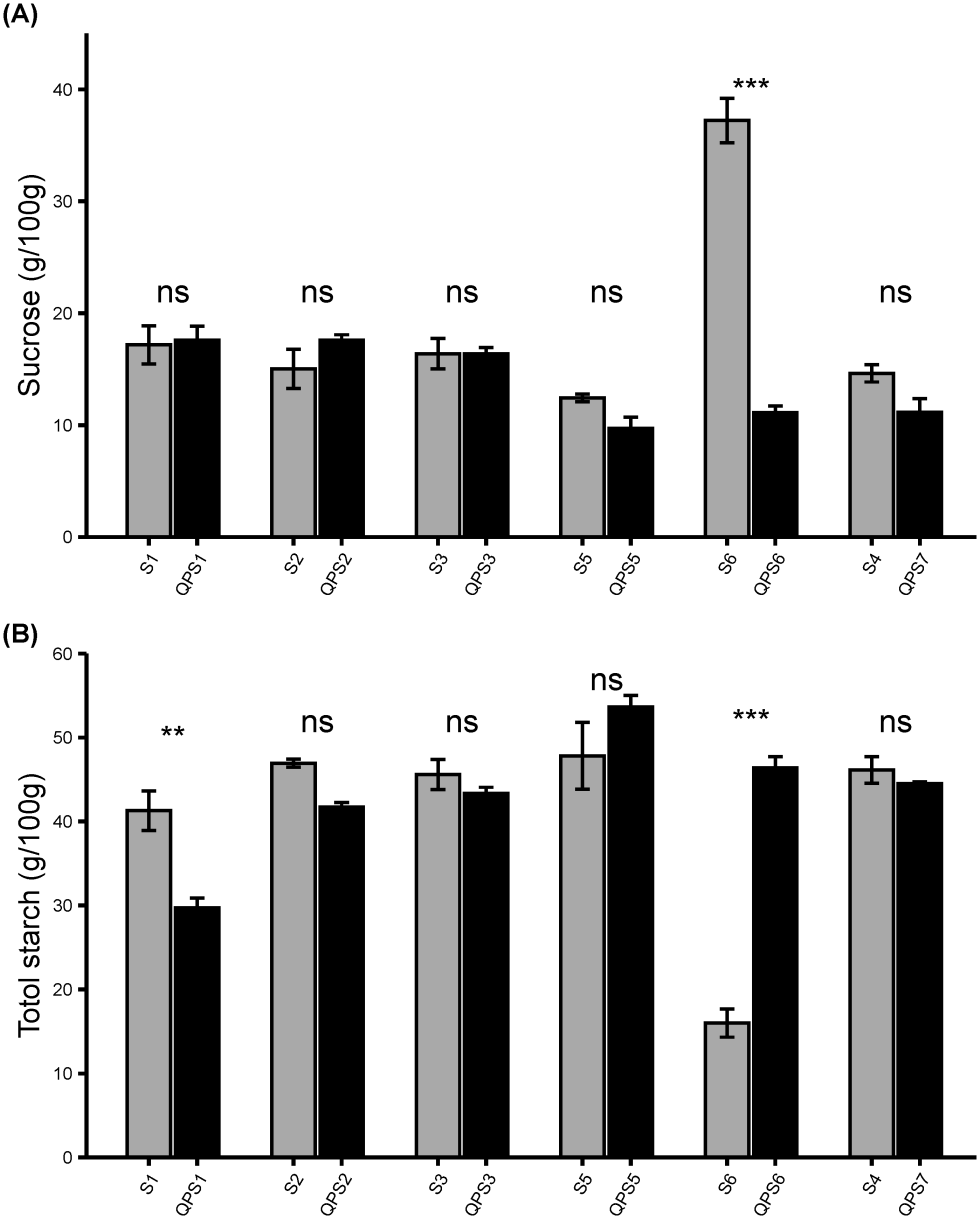
Determination of sugar content in F_5_ QPS inbreds. Sucrose and total starch were extracted from freshly harvested ears at 20 DAP. **(A)** sucrose amount (g/100g). **(B)** total starch amount (g/100g). Results are shown as mean ± SE (n = 6). Statistical difference between parental sweet corn, colored sweet corn inbreds and hybrids was determined using one-way ANOVA followed by Tukey’s HSD test. The asterisks show significant differences: **p < 0.01; ***p < 0.001, and ns, not significant.

**Figure 7:**
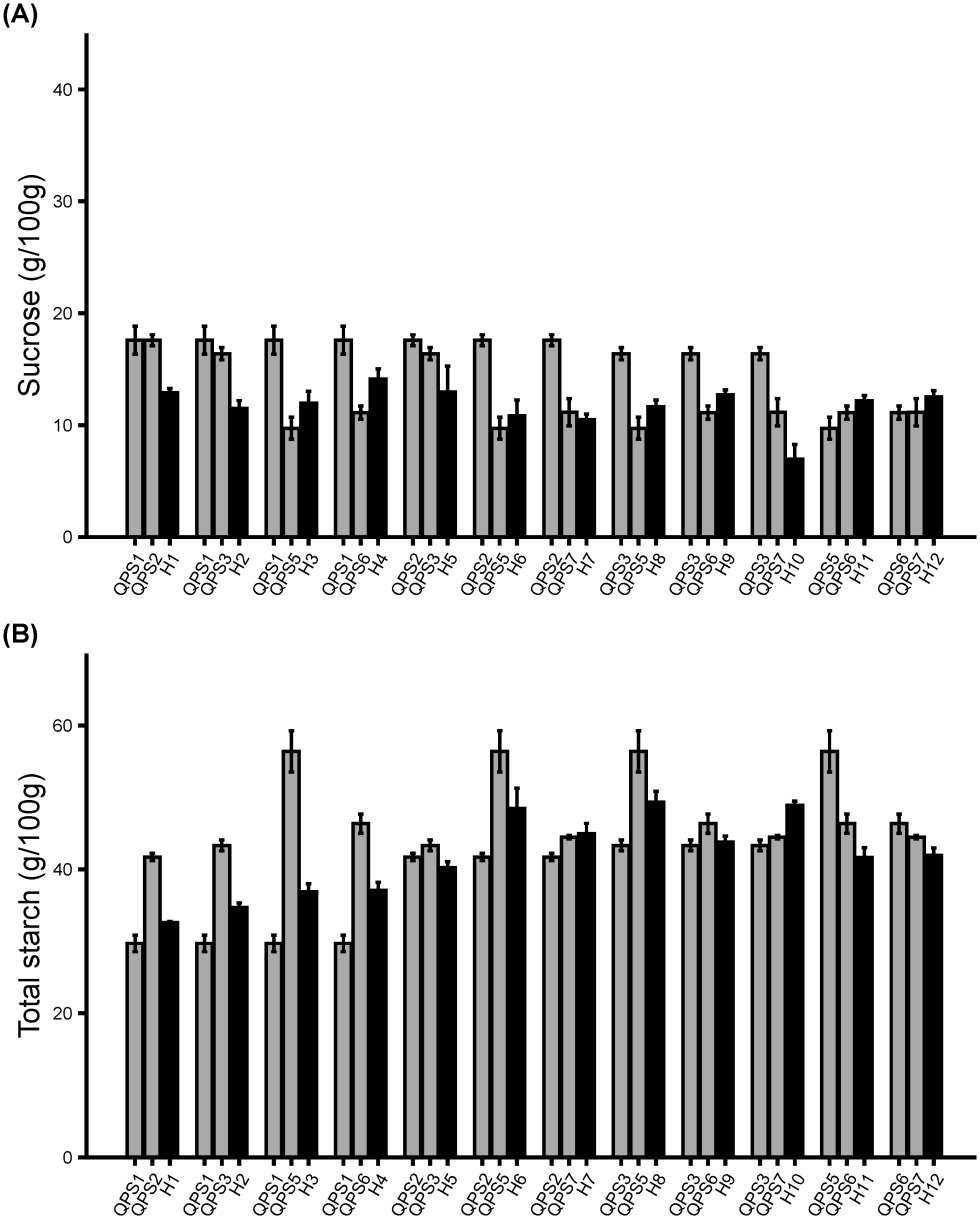
Determination of sugar content in F_2_ lines derived from F_1_ QPS hybrids. Sucrose and total starch were extracted from freshly harvested ears at 20 DAP. **(A)** sucrose amount (g/100g). **(B)** total starch amount. Results are shown as mean ± SE (n = 6).

To determine whether introgression of the *o2* mutation into wild-type sweet corn lines affected their sugar contents, the sucrose and total starch contents of the QPS inbred and hybrid lines were analyzed (**Figures 6**, **7**). Five of the six QPS inbred lines showed no significant change in the average sucrose content compared to their counterpart wild-type sweet corn lines (P>0.05) (**Figure 6A**). In contrast, QPS6 showed a significantly lower average sucrose amount than S6 (P<0.05). These five QPS lines were derived from *su1* sweet corn lines whereas QPS6 was derived from a *sh2* sweet corn line. Such observations suggest that the *o2* mutation had a negligible effect on the average sucrose content of *su1*-derived QPS inbred lines while heavily impacting that of *sh2*-derived QPS inbred lines. Overall, the majority of the QPS inbred lines had similar sucrose contents relative to their wild-type sweet corn counterparts. However, the sucrose content of QPS hybrid lines varies greatly regardless of their inbred parents’ performance (**Figure 7A**). Five QPS hybrids had lower sucrose than both corresponding QPS inbred parents, five QPS hybrids had higher sucrose than one of their corresponding QPS inbred parents, and only two QPS hybrids had higher sucrose than both of their corresponding QPS inbred parents. Seven of the 12 hybrid lines had higher sucrose than the average performance of their two inbred parents.

Sucrose is inversely related to starch which should be lower in sweet corn than non-sweet corn. Half of the QPS inbred lines showed no significant change in average seed total starch content compared with the sweet corn parents (P>0.05). In fact, two of the three QPS inbred lines showed a significantly lower average total starch compared to their corresponding wild-type sweet corn parents (**Figure 6B**). In contrast, one QPS inbred line showed a significantly higher average total starch compared to its wild-type sweet corn parent (P<0.05) (**Figure 6B**). These observations indicate that the total starch of the *su1*-derived QPS inbred lines were less affected by the *o2* mutation whereas the average total starch of *sh2*-derived QPS inbred were highly affected. Unlike the varying degree of the average sucrose amount in QPS hybrids, the average total starch of the QPS hybrids were lower than their mid-parent’s average total starch (**Figure 7B**). Thus, QPS hybrids maintained a more uniform total starch content lower than their corresponding parental inbred. Overall, the sucrose and total starch trends highlight that the *opaque-2* mutation did not interfere significantly with the pre-existing sweet corn *su1* mutation. However, the sweet corn *sh2* mutation was heavily affected but the effects were offset in the QPS hybrids through mid-parent heterosis. Conclusively, breeding *o2* mutation into wild-type sweet corn poses challenges to maintain seed sugar contents but careful selection can minimize adverse effects.

### QPS inbreds and hybrids maintained desirable sweetness and texture

**Figure 8:**
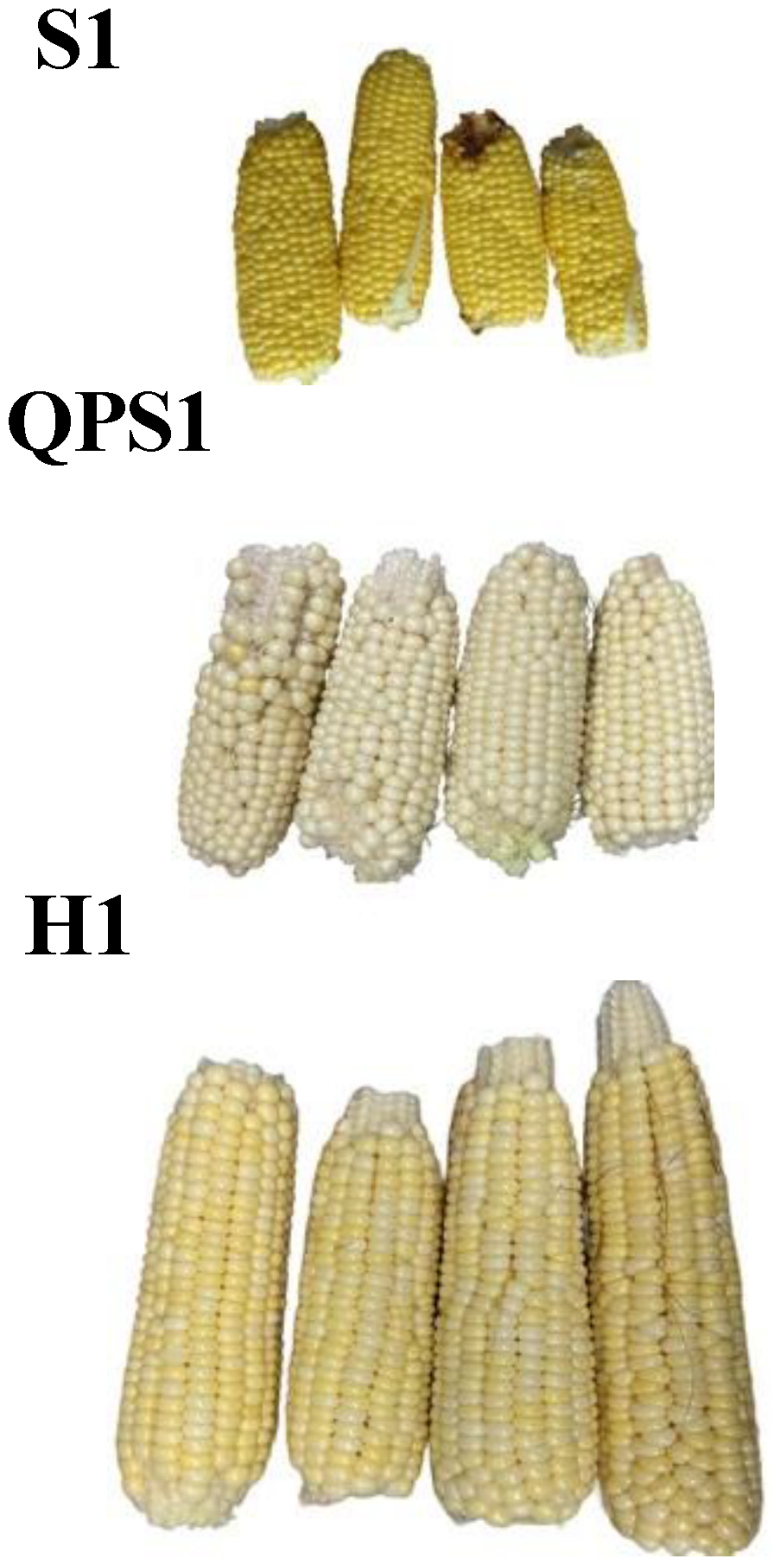
Evaluation of sweetness and texture among wild type sweet corn, QPS inbreds, and QPS hybrids. Freshly harvested ears (n = 4) were used for an in-field sensory testing of taste and texture. A consensus score ranging from 1 to 5 were assigned to indicate the overall kernel sweetness and texture.

To determine whether breeding *opaque-2* into sweet corn lines had adverse effects on the normal sweet corn sugary and tender phenotypes, taste and texture traits were evaluated in fresh QPS inbred and hybrid lines (**Figure 8**; **Supplementary Figures 13**, **S14**). A minimum of three hand pollinated, fresh wild-type sweet corn and QPS inbred lines were tested and scored for their taste and texture performance using an arbitrary scale of 1 to 5. A score of 3 was the absolute minimum requirement and anything below were discarded. Results revealed that all six QPS inbred lines had a minimum taste and texture score of 3 with some lines scoring as good as the wild-type lines (**Table 3**). Furthermore, the hybrids were also evaluated for their overall sensory performance in terms of taste and texture. Results showed that nine hybrids performed to the extent of their corresponding inbred parents while some showed diminished performance (**Table 4**). H1, H2, H3, H4, H5, H8, H9 hybrids performed well similarly to normal sweet corn. H7, H10, H11, and H12 hybrids scored less than normal but still acceptable taste and texture. Lastly, H6 and H7 hybrids produced the least desirable performance. In general, the majority of the QPS hybrids produced acceptable taste and texture. Slight reductions in taste and texture scores may be attributed to the residual dent corn QPM background in the QPS lines.

**Table 3:** Sweetness and texture comparison between sweet corn parents and QPS inbreds.

Lines	Taste score (1-5)		Texture score (1-5)	
	Sweet corn parent	QPS inbred	Sweet corn parent	QPS inbred
S_1_ & QPS_1_	5	3	4	5
S_2_ & QPS_2_	4	4	4	4
S_3_ & QPS_3_	4	3	4	4
S_4_ & QPS_7_	4	3	4	4
S_5_ & QPS_5_	4	3	4	4
S_6_ & QPS_6_	5	3	5	5

Taste and texture scores for sweet corn parents and QPS inbreds evaluated during Summer 2023. Freshly harvested ears (20 DAP) were scored for taste (1 = bland, 5 = sweet) and texture (1 = tough, 5 = tender).

**Table 4:** Sweetness and texture evaluation of QPS hybrids-derived F_2_ lines.

Hybrids	Taste score (1-5)	Texture score (1-5)	Phenotypic descriptions
H1	4	3	Tall plants, big fat ears, a little sweet with flavor.
H2	4	4	Tall plants, great fat ears, good fill. Bicolor (yellow & white). Good contender
H3	4	5	Tall plant and fat ears. Sweet and tender. Poor germination
H4	5	5	White huge ears and quite sweet and very tender. WINNING HYBRID
H5	4	4	Few ears have smut. Not a good candidate
H6	2	2	not sweet; not tender
H7	3	2	Tall plants and good ears, good flavor but not sweet
H8	5	4	Tall plants and big ears. Very sweet and tender.
H9	5	5	Tall plants and big ears. Very sweet and tender. WINNING HYBRID
H10	3	3	–
H11	3	3	Early, medium ears, not so tall(medium), mildly sweet and crispy tough
H12	3	3	Small ears, small kernels, bicolor, mildly sweet

Taste and texture scores for 12 QPS hybrids evaluated during Summer 2024. Freshly harvested ears (20 DAP) were scored for taste (1 = bland, 5 = sweet) and texture (1 = tough, 5 = tender).

### QPS hybrids maintained expected agronomics through heterosis effects

To confirm the heterosis effect of the QPS hybrids, the ear weight and ear length were measured in fresh QPS hybrids at the eating stage to examine the overall yield-related physical traits (**Figure 8**; **Supplementary Figure 14**). Results indicated that the average ear weight ranged from 176.17 to 256.23 g while the average ear length ranged from 137 to 188 mm (**Tables 5**, **6**). The ear weights were relatively equivalent across all the QPS hybrids except that H2 hybrid weighed significantly more than H10 (p=0.029), and H5 hybrid weighed significantly more than H10 (p=0.014) and H12 hybrids (p=0.043). In contrast, a third of all the ear lengths comparisons between the hybrids showed a significant difference (p<0.05) (**Supplementary Table 4**). In general, the ear weights did not vary much between hybrids and the ear lengths varied to some degree. These trends imply that while some hybrid ears were short, they compensated by increasing the kernel size and complete ear fill.

**Table 5:** Ear weight of QPS hybrids-derived F_2_ lines.

Hybrids	Weight (g)							
	Ear 1	Ear 2	Ear 3	Ear 4	Ear 5	Ear 6	Mean	*sd*
H1	254.4	252.1	222.3	–	–	–	242.9	17.9
H2	253.7	243.0	272.1	296.0	228.7	231.2	256.2	26.0
H3	218.7	219.6	220.8	190.9	226.9	166.0	219.7	23.8
H4	259.6	159.0	228.6	206.8	188.1	229.9	215.7	35.4
H5	244.9	256.2	220.5	264.0	265.5	247.0	240.5	16.6
H6	235.0	231.4	209.3	236.4	247.1	143.6	225.2	38.1
H7	223.8	226.1	261.6	205.9	245.1	166.0	237.1	33.2
H8	223.6	203.7	180.4	169.3	172.0	194.6	202.5	20.9
H9	247.4	177.0	180.2	267.8	226.3	213.0	201.5	36.2
H10	186.2	156.8	187.8	181.9	183.2	162.3	176.9	13.3
H11	203.5	153.7	171.4	199.5	118.1	192.3	176.2	32.8
H12	162.6	203.6	181.9	173.9	220.2	194.5	182.7	20.9

Freshly harvested (20 DAP) ears were husked and weighed in the summer of 2024.

**Table 6:** Ear length of QPS hybrids-derived F_2_ lines.

Hybrids	Ear Length (mm)							
	Ear 1	Ear 2	Ear 3	Ear 4	Ear 5	Ear 6	Mean	*sd*
H1	155	165	160	–	–	–	160	5
H2	150	145	140	145	120	130	138	11
H3	175	150	155	150	165	120	153	19
H4	180	120	180	140	150	150	153	23
H5	185	150	150	200	210	170	178	25
H6	190	180	190	200	180	140	180	21
H7	180	180	210	170	195	195	188	14
H8	190	185	160	180	155	160	172	15
H9	190	180	170	190	195	170	183	11
H10	110	130	160	145	140	135	137	17
H11	150	150	180	170	150	180	162	15
H12	145	170	170	165	170	175	166	11

Freshly harvested (20 DAP) ears were husked and measured in the summer of 2024.

## Discussion

### Sweet corn as a protein source

Globally, sweet corn is widely used as a fresh and canned vegetable and its demand has increased over the past two decades due to various factors including changing healthy dietary lifestyles (Sidahmed et al., 2025). Fresh sweet corn contains about 4% protein, which is notable when considering the kernels are more than 70% water. However, like other forms of maize, sweet corn is not a complete protein source since it lacks sufficient essential amino acids including lysine and tryptophan (Baveja et al., 2021; Mehta et al., 2021). This study clarifies the cause of the deficiency and demonstrates some scope for ameliorating it.

### High zein abundance at 20 DAP allows the *opaque-2* proteome rebalancing effect

Zein genes begin expression in the developing endosperm at around 10 DAP with the gamma zeins first followed by the alpha zeins (Woo et al., 2001). Different zein proteins have non-redundant roles in the hierarchical construction of ER protein bodies (Guo et al., 2013). Despite this early onset of zein transcript and protein accumulation, the relative accumulation of zeins with respect to other proteins (non-zeins) at mid-kernel development (prime eating stage in sweet corn) compared with that of mature kernels is ambiguous. At 20 DAP when sweet corn is normally consumed or processed, QPS lines showed a strong accumulation of zeins, the most abundant storage proteins in maize (Hartings et al., 2011). The reduction of the alpha-zeins in 20 DAP QPS lines confirmed that the effects of *o2* manifest similarly to mature kernels. Intriguingly, the analysis of the total protein in the resultant QPS inbreds showed a slight decrease in the protein concentration compared to the parental sweet corn although the reduction was only significant in two inbreds. Coupled with the complete knockdown of the 22-kDa α−zeins observed in the QPS inbreds, it appears that the zeins make up an even larger proportion of total kernel proteins in developing kernels than they do in mature kernels. This may indicate that early zein expression (Woo et al., 2001) at least in our inbreds, is accelerated with respect to the bulk of non-zein gene expression. However, the apparent protein concentration reductions seen in some QPS inbreds, were not observed in the QPS hybrids and in fact, protein concentrations were often increased compared with the inbred parents. The relative differences in total protein concentrations in the QPS inbreds and hybrids may be attributed to differences in rates of kernel maturation resulting from the heterosis effect.

Notwithstanding our observed differences between inbreds and hybrids, the relative consistency of protein concentrations between QPS and normal sweet corn lines further confirms that the increase of non-zein proteins, compensates the reduction of the alpha-zeins via the proteome rebalancing (Wu and Messing, 2014). This early accumulation of the alpha zeins allowed *o2* to cause a proteome rebalancing effect, hence increasing lysine content. The demonstrated lysine increase in sweet corn, shows for the first time that zein abundance at 20 DAP is indeed, more than sufficient for an *o2* induced proteome rebalancing effect. The reduction in alpha zeins causes proteome rebalancing within the endosperm in monocots (Li et al., 2018; Parsons et al., 2020; Zhao et al., 2023; Hurst et al., 2024). Similar processes happen within the embryo in dicot seeds (Herman, 2014), The nature of proteome rebalancing control is under investigation and is thought to be controlled largely at the level of translation (Bagaza et al., 2024).

### Zein decrease causes increased lysine as well as some collateral amino acid effects

The non-zein proteins collectively contain more lysine and tryptophan (Wu and Messing, 2014; Devi et al., 2024) which is the basis for the reported increase in the protein-bound lysine in the QPS lines. Furthermore, alpha zeins are rich in proline, alanine, and leucine amino acids (Tatham et al., 1993). Analysis of amino acids profiles revealed significant reductions in these amino acids (**Supplementary Figures 4**, **8**, **S9**) due to the reduction of alpha zeins accumulation.

Besides lysine, several other essential amino acids such as isoleucine, leucine, phenylalanine, methionine, and threonine were affected by *opaque-2* introgression. Our findings indicated that the protein-bound fractions of isoleucine, leucine, phenylalanine reduced in all six QPS inbred lines and also the protein-bound fraction of methionine reduced in four QPS inbred lines in comparison to the parental sweet corn. In contrast, two QPS inbred lines showed increased protein-bound methionine and five QPS inbred lines showed increased protein-bound threonine contents relative to their parental sweet corn lines. The findings presented by Hurst et al. (2024) support these observations. Hurst et al. reported that alpha zeins, which are regulated by the OPAQUE-2 (O2) transcription factor, contain isoleucine, leucine, and phenylalanine. Thus, we would expect *o2* mutation to cause a reduction of those amino acids. Moreover, O2 regulates the 15-kDa β−zeins, which are rich in methionine. Again, the *o2* mutation would lead to a reduction in the fraction of protein-bound methionine which agrees with our findings. In addition, Hurst et al. (2024) suggested that the increase in free amino acid may be attributed to the *o2* mutation since the O2 regulates enzymes such as Lysine Ketoglutarate reductase (LKR) and aspartate kinase 2 (Ask2) which regulates and catabolizes free amino acids in maize (Wang et al., 2001). Therefore, the loss of function in O2 transcription factor would result in increased accumulation of free amino acids.

With the alpha zeins accounting for 70% of maize storage proteins (Xing et al., 2023), it is expected that their reduced accumulation will result in reduced utilization of free amino acids and increases in free amino acid pools. Protein degradation and synthesis strongly affect the direction of conversion between free and protein-bound amino acids; increase in the free amino acids were observed following the protein degradation in Arabidopsis (Hildebrandt, 2018). These findings are consistent with our amino acid results. An inverse interaction between the free and protein-bound amino acids was observed in Ala, His, Ile, Leu, Lys, Phe, Pro, Ser, Tyr, and Val (**Supplementary Table 5**). These results indicate that free amino acids increased while PBAA decreased. However, the increasing trend shown in most free amino acids may have little relevance. The ratio of the PBAA to FAA contents was at least 10X higher (**Supplementary Table 3**) such that increases in free amino acid may have little effect in offsetting the PBAA reductions. Furthermore, the relevance of the reduction in some protein-bound amino acids may depend on how limiting they are in human diets. The limiting amino acids in humans and animals, particularly non-ruminants include lysine and tryptophan (primary colimiting AA); isoleucine, methionine, threonine, and possibly valine (Lewis et al., 1982; Tang et al., 2021; Devi et al., 2023, 2024). Within the QPS lines, there were no significant adverse impacts on the PB threonine, methionine, and valine, (**Supplementary Figures 7**-**S9**). However, the collateral effects of *o2* were observed in the secondary limiting isoleucine (**Supplementary Figure 4**). In general, the collateral effects of *o2* on the limiting amino acids were minimal and may be acceptable given its positive implication on lysine content.

### *Sugary-1* and *shrunken-2* as substrates for QPS

In this study, we have shown that the *opaque-2* mutation can be used to improve the protein quality of sweet corn using a conventional breeding approach. Similar to a previous study (Ren et al., 2018), the introgression of the *opaque-2* mutation into popcorn varieties led to the development of high-lysine popcorn varieties (QPP) with increased protein-bound and free lysine. However, though the popcorn study succeeded, the introgression of *opaque-2* mutation has some adverse alterations on the physical and physiological traits most notably slightly reduced popcorn flake expansion. Negative effects were also observed in QPS. It has been observed that the sucrose content decreased, and starch content increased in comparison to the original sucrose and starch contents in parental sweet corn lines. The alterations in sugar and starch contents were more severe in QPS6 than the other QPS inbred lines. This phenomenon may be attributed to the differences in the mutations present in those varieties. QPS6 was derived from a *shrunken-2* (*sh2)* sweet corn parent whereas the rest of the QPS inbreds were derived from a *sugary-1* (*su1)* sweet corn parent. The *sh2* mutants reduce the proportion of carbohydrates and increase the proportion of sugars in the maize endosperm while the *su1* mutants only changes the ratio of sugar contents to the proportion of carbohydrates and other water-soluble polysaccharides (Dodson-Swenson and Tracy, 2015; Mehta et al., 2021). Thus, *sh2* varieties contain higher sugar content and are sweeter than *su1* varieties (Hu et al., 2021). The *opaque-2* mutation only affects the protein accumulation and not sugar accumulation. However, it was reported that O2 transcription factor activates the expression of *AGPL2* which regulates the starch accumulation in the sweet corn endosperm (Chen et al., 2026). In addition, Chen et al. found that *O2*-RNAi kernels displayed reduced AGPase activity, which similarly to sh2 mutation in sweet corn that inhibit the AGPase activity leading to increase sugar content, would reduce the starch accumulation and possibly increase the sugar content. Given these *O2*-RNAi findings, we would expect the *o2* QPS inbreds to exhibit similar low starch and higher sugar contents. Hence, the cause for the increased starch content in some QPS lines is not clear and we can only speculate that the reduction of sugar and increased total starch in QPS6 inbreds may be attributed to the QTL/modifier genes from the dent corn QPM. Another explanation for increased starch in *sh2* derived QPS may be related to rebalancing of carbon resources. Despite the AGPase mutation, starch accumulation is not completely blocked in *sh2* mutant kernels. In the context of reduced zein accumulation, and in addition to proteome rebalancing to non-zeins, it is possible that rebalancing of carbon flux towards this residual starch accumulation may be increased.

A study showed that the *opaque-2* sweet corn displayed a varying level of total sugars even though the sweetness remained within the normal range (Jompuk et al., 2020). Moreover, another study hypothesized that the reported minor variation in sweetness in the *opaque2* sweet corn may be attributed to various QTL/modifier genes that affected the sugar accumulation (Mehta et al., 2020) These observations agree with our results where the sugar and total starch contents (**Figures 6**, **7**) varied among the QPS inbreds, but the preliminary scoring of sweetness remained within the satisfactory ranges (**Table 3**).

Given the starchy background of the QPM dent corn used in the cross with sweet corn, efforts were made to maintain kernel sweetness, flavor, and tenderness which are main sweet corn qualities (Dodson-Swenson and Tracy, 2015). The sucrose content is the main indicator of the sweetness which also determines the flavor, and the proportion of water-soluble and insoluble polysaccharides controls the kernel hardness which controls the texture (mouth feel). The preliminary evaluation of kernel eating qualities played a key role in the early-stage selection of the QPS lines comparable to sweet corn parents. While the testing panel size was limited to three people, the use of minimum three ears per QPS line and the independent taste scoring allowed us to identify and select best QPS lines that possessed satisfactory sweet corn eating qualities vital for consumer-preferences. Importantly, this selection was implemented prior to *o2* confirmation; therefore, ensuring that QPS lines that were advanced to next generation exhibited both acceptable sweet corn qualities in addition to *o2* trait. With this selection approach, we ensured that not only QPS lines achieved improved nutritional value but also maintained market potential.

In conclusion, the introgression of the *o2* mutation into sweet corn lines resulted in the production of six QPS inbreds and 12 QPS hybrids. The SDS-PAGE profiling of the zeins indicated that they strongly accumulate by 20 DAP; thus, allowing *o2* to initiate a proteome rebalancing effect resulting in high lysine content in developing sweet corn. Biochemical analysis of sugar contents in QPS inbreds and hybrids revealed that the QPS lines of *su1* type had no significant collateral changes in sucrose and starch contents compared with the QPS lines of *sh2* type. The *sh2* sweet corn has substantially higher sugar and lower starch contents than *su1* sweet corn; therefore, the *sh2* types are more likely to register any sugar profile disturbance associated with the increased starch background from the initial crossing to the highly starchy QPM dent corn. Even so, the kernel eating qualities of *sh2*-derived QPS lines were equivalent to that of *su1* QPS lines. By incorporating the selection for acceptable sweet corn eating qualities along with the *o2* trait, we recovered high lysine sweet corn lines with sugar contents comparable to parental sweet corns but with enhanced essential lysine and potential marketability.

## Data Availability

The original contributions presented in the study are included in the article/**Supplementary Material**. Further inquiries can be directed to the corresponding author.

## Supplementary material

Supplementary Figure 1 Visual phenotyping of the *o2* mutation in parental sweet corn and QPS lines.

Supplementary Figure 2 SDS-PAGE confirmation of *o2* in QPS introgression. **(A)** F_5_ QPS inbreds. **(B)** F_2_ lines derived from F_1_ QPS hybrids.

Supplementary Figure 3 SDS-PAGE confirmation of *o2* in F_2_ lines derived from F_1_ QPS hybrids.

Supplementary Figure 4 Profiling amino acids in six F_5_ QPS inbreds and 12 F_2_ lines derived from F_1_ QPS hybrids. Fresh ears harvested at 20 DAP were used for amino acid extraction. **(A)** Protein-bound isoleucine in F_5_ QPS inbreds. **(B)** Free isoleucine in F_5_ QPS inbreds. **(C)** Protein-bound isoleucine in F_2_ lines derived from F_1_ QPS hybrids. **(D)** Free isoleucine in F_2_ lines derived from F_1_ QPS hybrids. Results are shown as mean ± SE for n = 3–6 biological replicates. Significance was determined by independent t-test comparison between parental sweet corn as control (light yellow) and QPS inbreds as treatment (black). *p < 0.05; **p < 0.01; ***p < 0.001.

Supplementary Figure 5 Profiling amino acids in six F_5_ QPS inbreds and 12 F_2_ lines derived from F_1_ QPS hybrids. Fresh ears harvested at 20 DAP were used for amino acid extraction. **(A)** Protein-bound leucine in F_5_ QPS inbreds. **(B)** Free leucine in F_5_ QPS inbreds. **(C)** Protein-bound leucine in F_2_ lines derived from F_1_ QPS hybrids. **(D)** Free leucine in F_2_ lines derived from F_1_ QPS hybrids. Results are shown as mean ± SE for n = 3–6 biological replicates. Significance was determined by independent t-test comparison between parental sweet corn as control (light yellow) and QPS inbreds as treatment (black). *p < 0.05; **p < 0.01; ***p < 0.001.

Supplementary Figure 6 Profiling amino acids in six F_5_ QPS inbreds and 12 F_2_ lines derived from F_1_ QPS hybrids. Fresh ears harvested at 20 DAP were used for amino acid extraction. **(A)** Protein-bound phenylalanine in F_5_ QPS inbreds. **(B)** Free phenylalanine in F_5_ QPS inbreds. **(C)** Protein-bound phenylalanine in F_2_ lines derived from F_1_ QPS hybrids. **(D)** Free phenylalanine in F_2_ lines derived from F_1_ QPS hybrids. Results are shown as mean ± SE for n = 3–6 biological replicates. Significance was determined by independent t-test comparison between parental sweet corn as control (light yellow) and QPS inbreds as treatment (black). *p < 0.05; **p < 0.01; ***p < 0.001.

Supplementary Figure 7 Profiling amino acids in six F_5_ QPS inbreds and 12 F_2_ lines derived from F_1_ QPS hybrids. Fresh ears harvested at 20 DAP were used for amino acid extraction. **(A)** Protein-bound methionine in F_5_ QPS inbreds. **(B)** Free methionine in F_5_ QPS inbreds. **(C)** Protein-bound methionine in F_2_ lines derived from F_1_ QPS hybrids. **(D)** Free methionine in F_2_ lines derived from F_1_ QPS hybrids. Results are shown as mean ± SE for n = 3–6 biological replicates. Significance was determined by independent t-test comparison between parental sweet corn as control (light yellow) and QPS inbreds as treatment (black). *p < 0.05; **p < 0.01; ***p < 0.001.

Supplementary Figure 8 Profiling amino acids in six F_5_ QPS inbreds and 12 F_2_ lines derived from F_1_ QPS hybrids. Fresh ears harvested at 20 DAP were used for amino acid extraction. **(A)** Protein-bound threonine in F_5_ QPS inbreds. **(B)** Free threonine in F_5_ QPS inbreds. **(C)** Protein-bound threonine in F_2_ lines derived from F_1_ QPS hybrids. **(D)** Free threonine in F_2_ lines derived from F_1_ QPS hybrids. Results are shown as mean ± SE for n = 3–6 biological replicates. Significance was determined by independent t-test comparison between parental sweet corn as control (light yellow) and QPS inbreds as treatment (black). *p < 0.05; **p < 0.01; ***p < 0.001.

Supplementary Figure 9 Analysis of essential and conditionally essential protein-bound amino acids (g/100 total dry weight). Fresh 20 DAP ears were used for analysis (n =6).

Supplementary Figure 10 Analysis of conditionally essential and non-essential protein-bound amino acids (g/100 total dry weight). Fresh 20 DAP ears were used for analysis (n =6).

Supplementary Figure 11 Analysis of essential and conditionally essential free amino acids (g/100 total dry weight). Fresh 20 DAP ears were used for analysis (n =6).

Supplementary Figure 12 Analysis of conditionally essential and non-essential free amino acids (g/100 total dry weight). Fresh 20 DAP ears were used for analysis (n =6).

Supplementary Figure 13 Evaluation of taste and texture sensory performance among wild type sweet corn and QPS inbreds. Freshly harvested ears (n = 4) were used for an in-field sensory testing of taste and texture. A consensus score ranging from 1 to 5 was assigned to indicate the overall kernel sweetness and texture performance.

Supplementary Figure 14 Evaluation of taste and texture sensory performance of F_2_ lines derived from F_1_ QPS hybrids. Freshly harvested ears (n = 4) were used for an in-field sensory testing of taste and texture. A consensus score ranging from 1 to 5 was assigned to indicate the overall kernel sweetness and texture performance.

Supplementary Table 1 Mean and *sd* values of protein-bound amino acids (g/100g dry weight) in parental sweet corn, QPS inbreds and hybrids.

Supplementary Table 2 Mean and *sd* values of free amino acids (g/100g dry weight) in parental sweet corn, QPS inbreds and hybrids.

Supplementary Table 3 PBAA/FAA ratios in QPS and parental sweet corn.

Supplementary Table 4 Ear weight and length comparison between F_2_ lines derived from F_1_ QPS hybrids.

Supplementary Table 5 QPS/S ratios for free and protein-bound amino acids.
