# Supplementary material for: Opaque-2 induced zein reduction and lysine increase suggest a route to quality protein sweet corn
Source: Front Plant Sci. 2026 May 14;17:1814115. doi: 10.3389/fpls.2026.1814115 (PMC13216212; doi:10.3389/fpls.2026.1814115)
Supplement: Supplementary Figure 1 — Visual phenotyping of the o2 mutation in parental sweet corn and QPS lines. [file Image1.pdf]

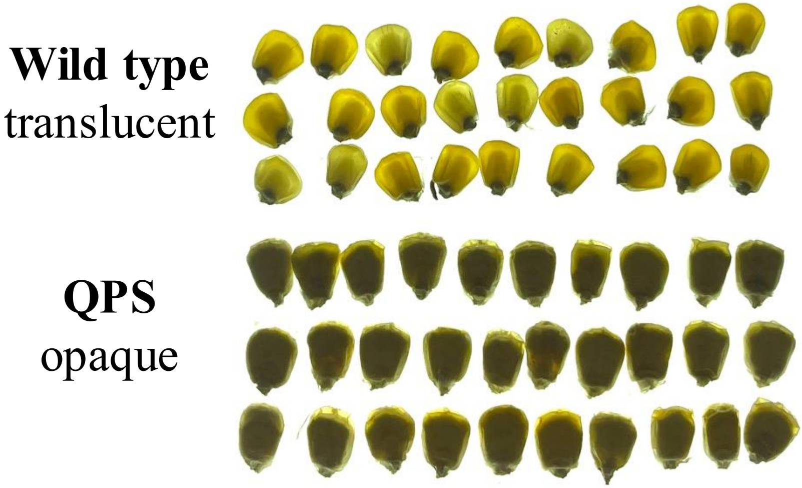

Supplementary Figure S1| Visual phenotyping of the o2 mutation in parental sweet corn and QPS lines.

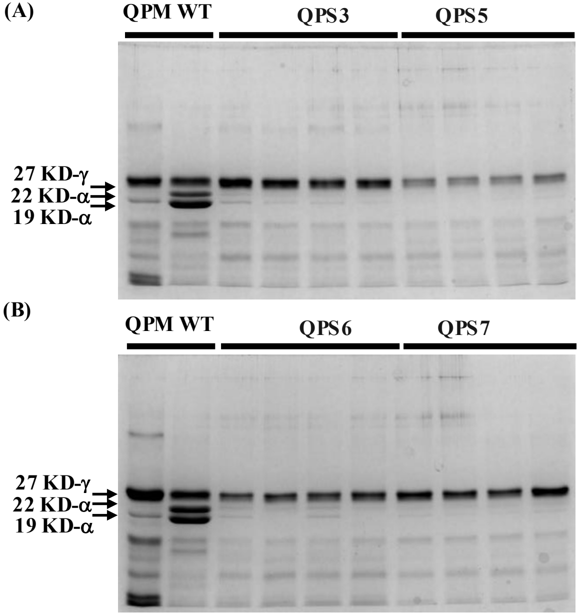
(A)
QPM WT
QPS3
QPS5
27 KD-γ
22 KD-α
19 KD-α
(B)
QPM WT
QPS6
QPS7
27 KD-γ
22 KD-α
19 KD-α

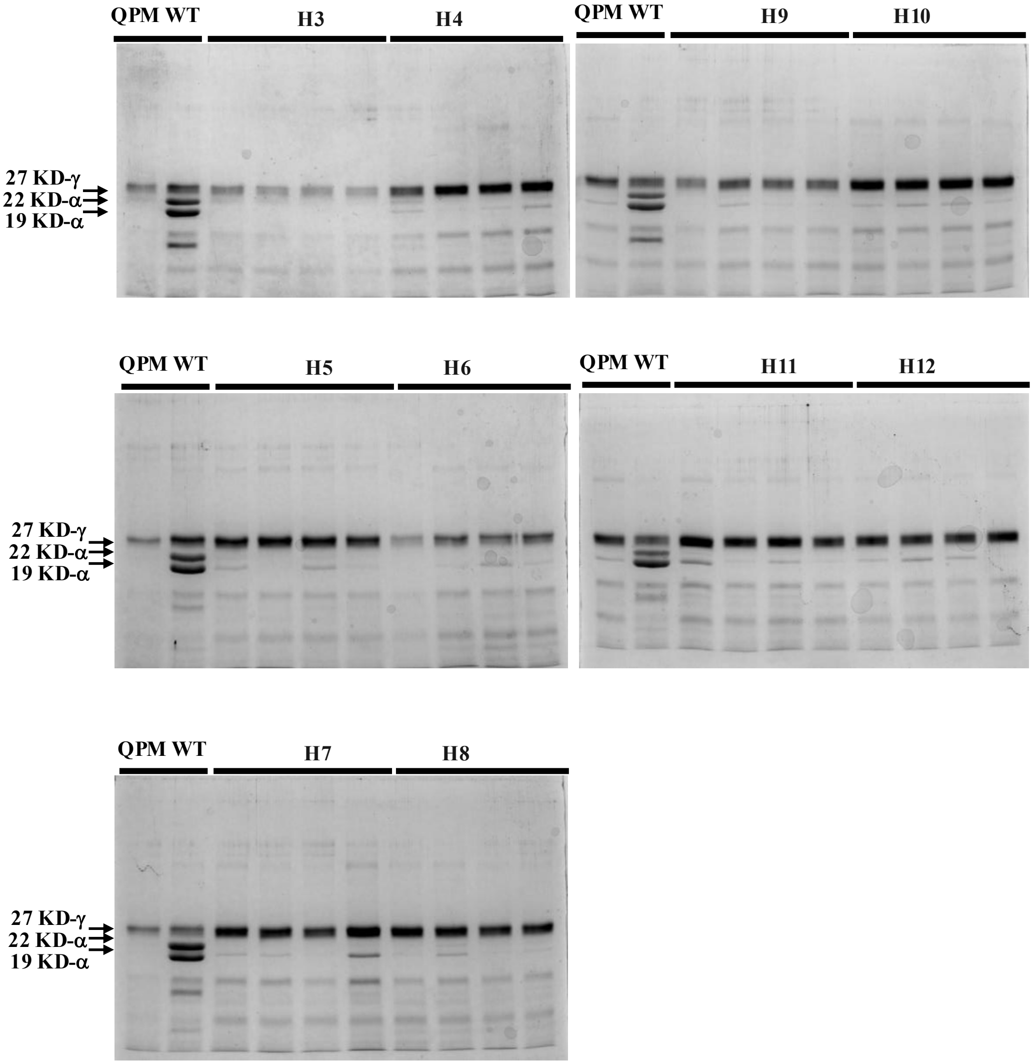
QPM WT
H3
H4
27 KD-γ
22 KD-α
19 KD-α
QPM WT
H9
H10
QPM WT
H5
H6
27 KD-γ
22 KD-α
19 KD-α
QPM WT
H11
H12
QPM WT
H7
H8
27 KD-γ
22 KD-α
19 KD-α

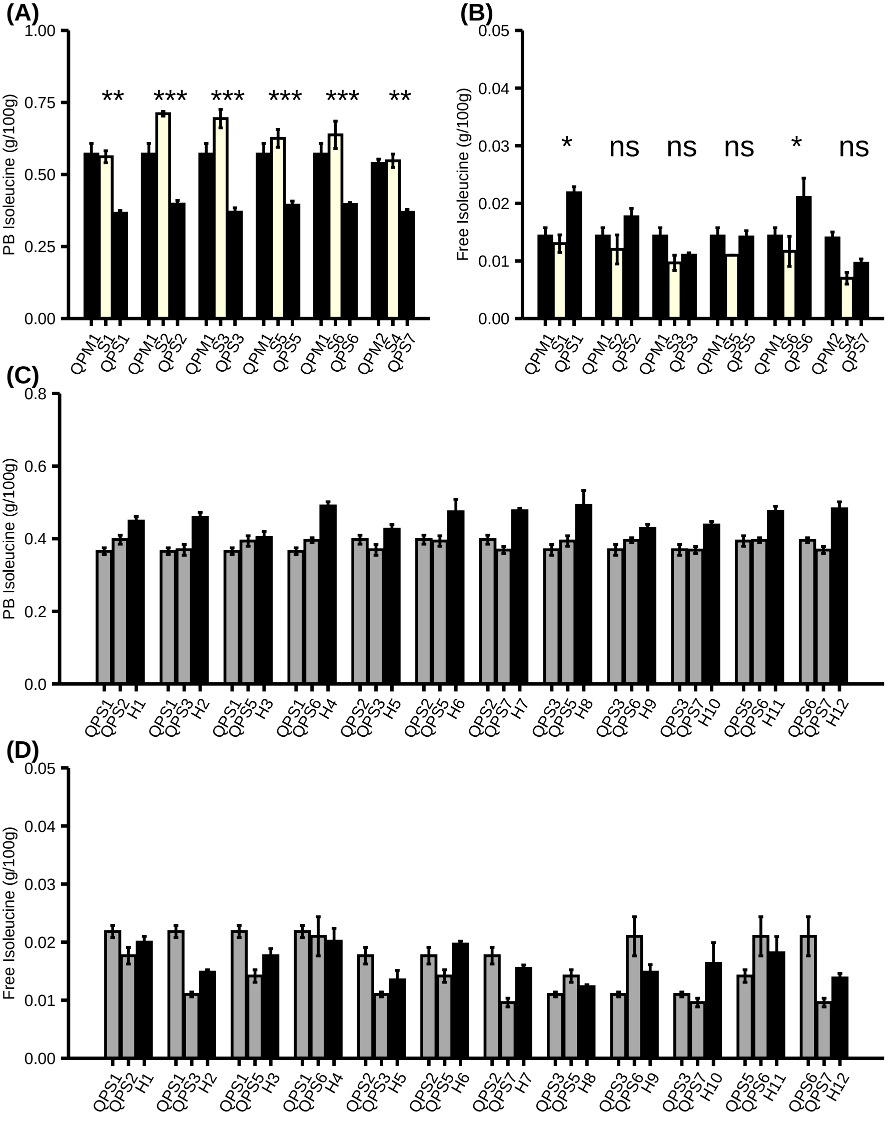

(A)
PB Isoleucine (g/100g)
1.00
0.75
0.50
0.25
0.00
** *** *** *** *** **
QPM1 S1 QPS1 QPM1 S2 QPS2 QPM1 S3 QPS3 QPM1 S5 QPS5 QPM1 S6 QPS6 QPM2 S4 QPS7
(B)
Free Isoleucine (g/100g)
0.05
0.04
0.03
0.02
0.01
0.00
* ns ns ns * ns
QPM1 S1 QPS1 QPM1 S2 QPS2 QPM1 S3 QPS3 QPM1 S5 QPS5 QPM1 S6 QPS6 QPM2 S4 QPS7
(C)
PB Isoleucine (g/100g)
0.8
0.6
0.4
0.2
0.0
QPS1 QPS2 H1 QPS1 QPS3 H2 QPS1 QPS5 H3 QPS1 QPS6 H4 QPS2 QPS3 H5 QPS2 QPS5 H6 QPS2 QPS7 H7 QPS3 QPS5 H8 QPS3 QPS6 H9 QPS3 QPS7 H10 QPS5 QPS6 H11 QPS6 QPS7 H12
(D)
Free Isoleucine (g/100g)
0.05
0.04
0.03
0.02
0.01
0.00
QPS1 QPS2 H1 QPS1 QPS3 H2 QPS1 QPS5 H3 QPS1 QPS6 H4 QPS2 QPS3 H5 QPS2 QPS5 H6 QPS2 QPS7 H7 QPS3 QPS5 H8 QPS3 QPS6 H9 QPS3 QPS7 H10 QPS5 QPS6 H11 QPS6 QPS7 H12

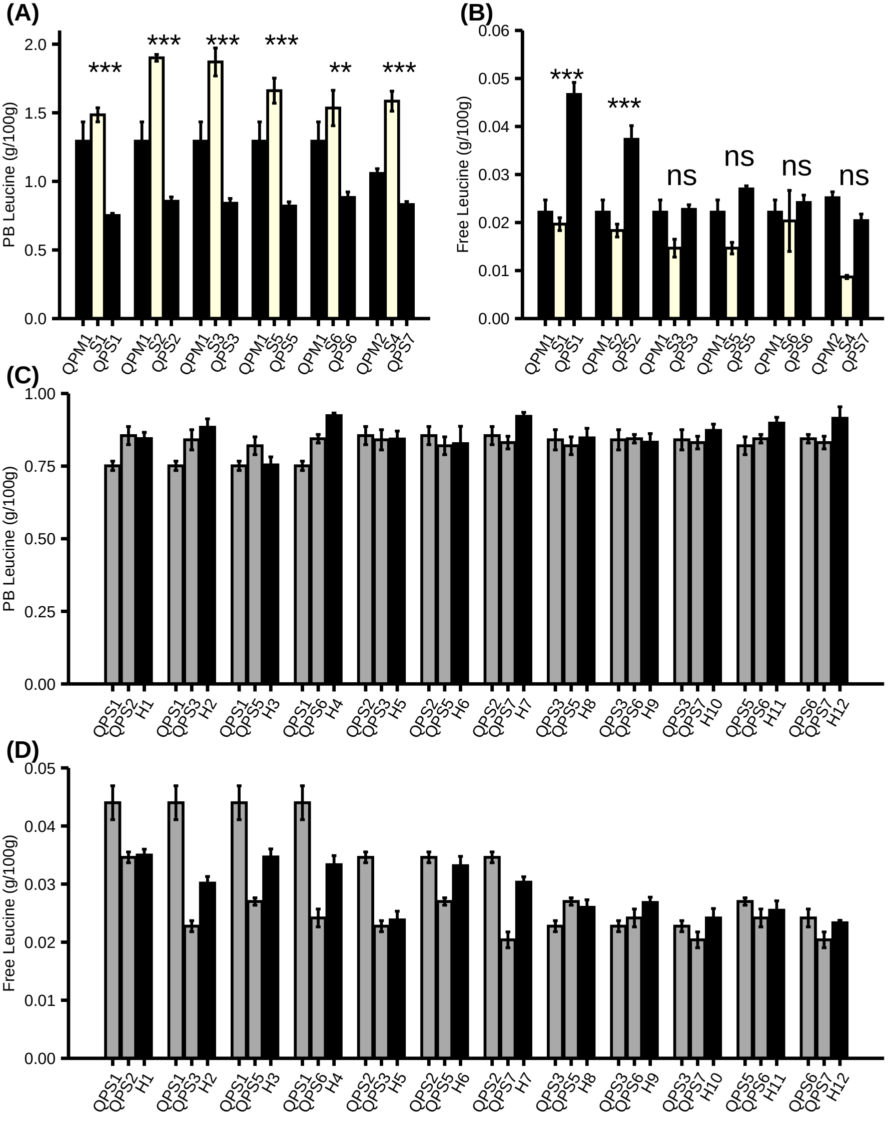

(A)
PB Leucine (g/100g)
0.0
0.5
1.0
1.5
2.0
***
***
***
***
**
***
QPM1 S1 QPS1
QPM1 S2 QPS2
QPM1 S3 QPS3
QPM1 S5 QPS5
QPM1 S6 QPS6
QPM2 S4 QPS7
(B)
Free Leucine (g/100g)
0.00
0.01
0.02
0.03
0.04
0.05
0.06
***
***
ns
ns
ns
ns
QPM1 S1 QPS1
QPM1 S2 QPS2
QPM1 S3 QPS3
QPM1 S5 QPS5
QPM1 S6 QPS6
QPM2 S4 QPS7
(C)
PB Leucine (g/100g)
0.00
0.25
0.50
0.75
1.00
QPS1 QPS2 H1
QPS1 QPS3 H2
QPS1 QPS5 H3
QPS1 QPS6 H4
QPS2 QPS3 H5
QPS2 QPS5 H6
QPS2 QPS7 H7
QPS3 QPS5 H8
QPS3 QPS6 H9
QPS3 QPS7 H10
QPS5 QPS6 H11
QPS6 QPS7 H12
(D)
Free Leucine (g/100g)
0.00
0.01
0.02
0.03
0.04
0.05
QPS1 QPS2 H1
QPS1 QPS3 H2
QPS1 QPS5 H3
QPS1 QPS6 H4
QPS2 QPS3 H5
QPS2 QPS5 H6
QPS2 QPS7 H7
QPS3 QPS5 H8
QPS3 QPS6 H9
QPS3 QPS7 H10
QPS5 QPS6 H11
QPS6 QPS7 H12

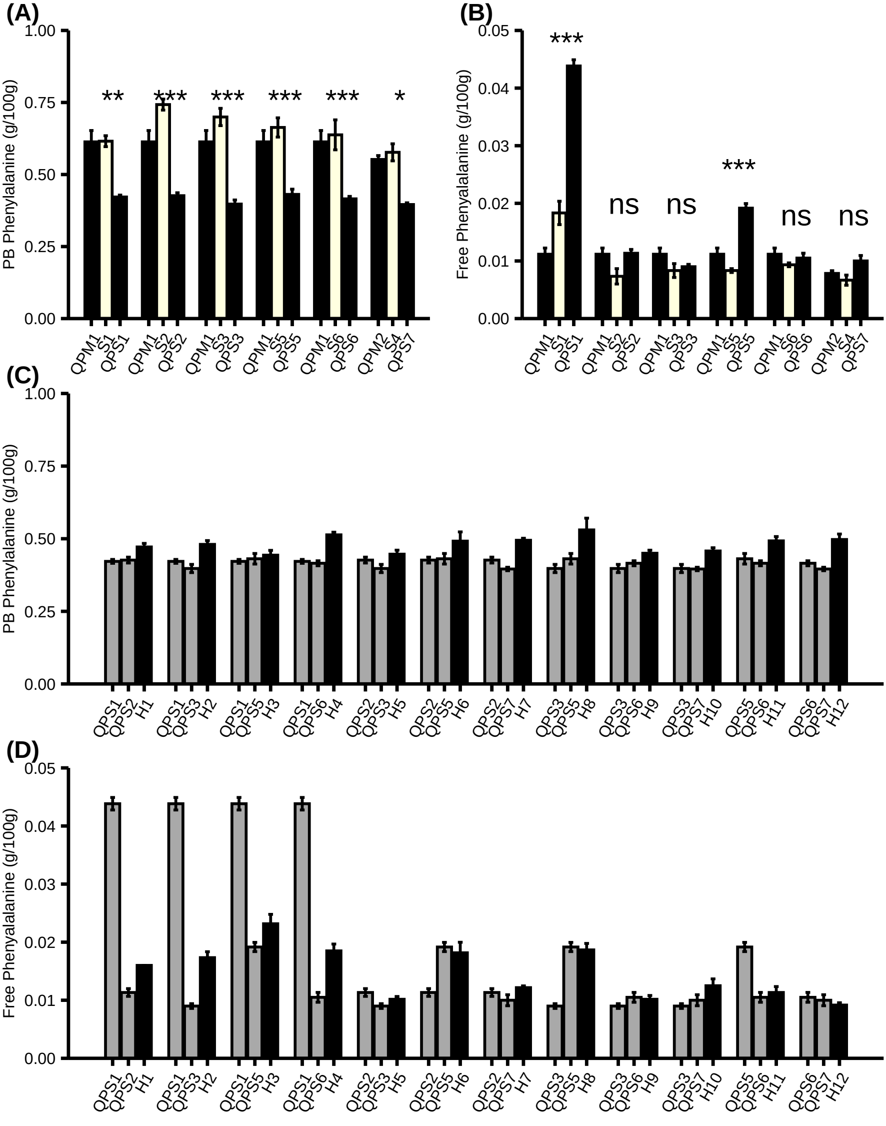
(A)
PB Phenylalanine (g/100g)
1.00
0.75
0.50
0.25
0.00
** *** *** *** *** *
QPM1 S1 QPS1 QPM1 S2 QPS2 QPM1 S3 QPS3 QPM1 S5 QPS5 QPM1 S6 QPS6 QPM2 S4 QPS7
(B)
Free Phenylalanine (g/100g)
0.05
0.04
0.03
0.02
0.01
0.00
*** ns ns *** ns ns
QPM1 S1 QPS1 QPM1 S2 QPS2 QPM1 S3 QPS3 QPM1 S5 QPS5 QPM1 S6 QPS6 QPM2 S4 QPS7
(C)
PB Phenylalanine (g/100g)
1.00
0.75
0.50
0.25
0.00
QPS1 QPS2 H1 QPS1 QPS3 H2 QPS1 QPS5 H3 QPS1 QPS6 H4 QPS2 QPS3 H5 QPS2 QPS5 H6 QPS2 QPS7 H7 QPS3 QPS5 H8 QPS3 QPS6 H9 QPS3 QPS7 H10 QPS5 QPS6 H11 QPS6 QPS7 H12
(D)
Free Phenylalanine (g/100g)
0.05
0.04
0.03
0.02
0.01
0.00
QPS1 QPS2 H1 QPS1 QPS3 H2 QPS1 QPS5 H3 QPS1 QPS6 H4 QPS2 QPS3 H5 QPS2 QPS5 H6 QPS2 QPS7 H7 QPS3 QPS5 H8 QPS3 QPS6 H9 QPS3 QPS7 H10 QPS5 QPS6 H11 QPS6 QPS7 H12

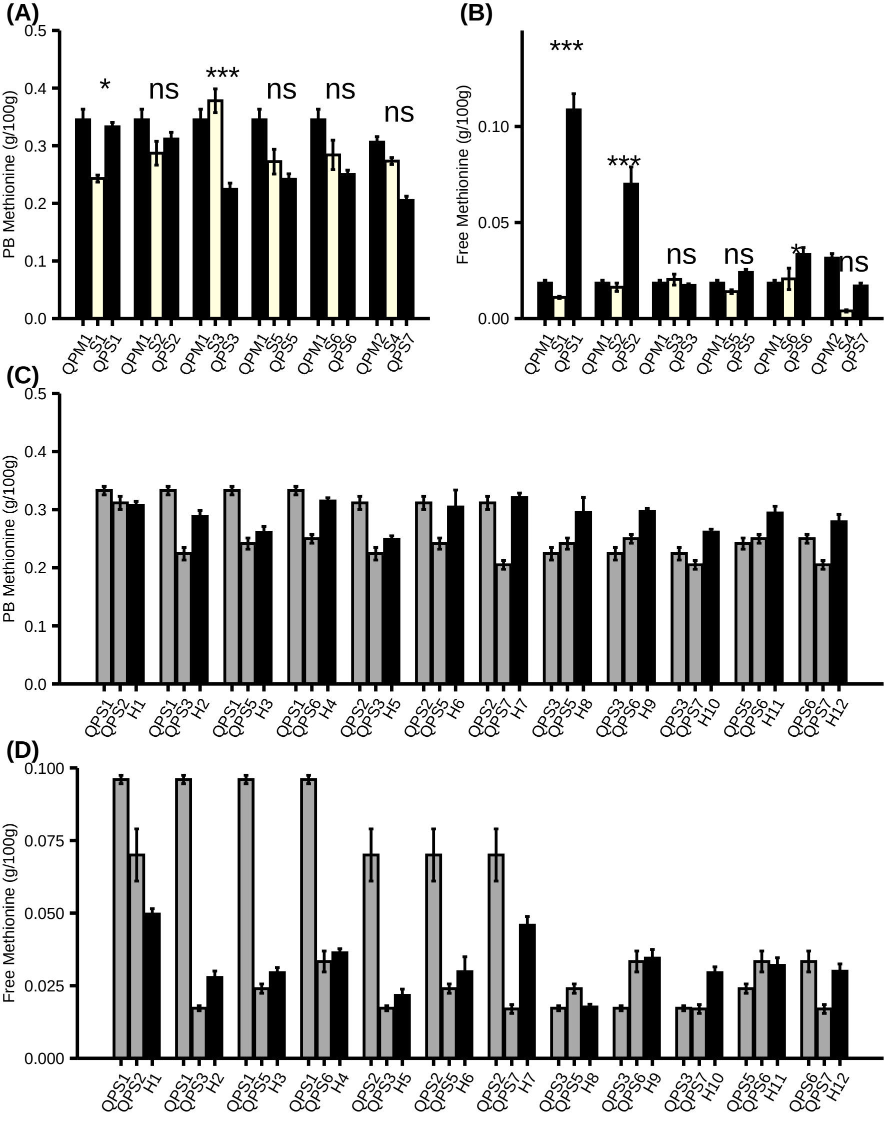

(A)
PB Methionine (g/100g)
0.0
0.1
0.2
0.3
0.4
0.5
*
ns
***
ns
ns
ns
QPM1 S1 QPS1
QPM1 S2 QPS2
QPM1 S3 QPS3
QPM1 S5 QPS5
QPM1 S6 QPS6
QPM2 S4 QPS7
(B)
Free Methionine (g/100g)
0.00
0.05
0.10
***
***
ns
ns
*
ns
QPM1 S1 QPS1
QPM1 S2 QPS2
QPM1 S3 QPS3
QPM1 S5 QPS5
QPM1 S6 QPS6
QPM2 S4 QPS7
(C)
PB Methionine (g/100g)
0.0
0.1
0.2
0.3
0.4
0.5
QPS1 QPS2 H1
QPS1 QPS3 H2
QPS1 QPS5 H3
QPS1 QPS6 H4
QPS2 QPS3 H5
QPS2 QPS5 H6
QPS2 QPS7 H7
QPS3 QPS5 H8
QPS3 QPS6 H9
QPS3 QPS7 H10
QPS5 QPS6 H11
QPS6 QPS7 H12
(D)
Free Methionine (g/100g)
0.000
0.025
0.050
0.075
0.100
QPS1 QPS2 H1
QPS1 QPS3 H2
QPS1 QPS5 H3
QPS1 QPS6 H4
QPS2 QPS3 H5
QPS2 QPS5 H6
QPS2 QPS7 H7
QPS3 QPS5 H8
QPS3 QPS6 H9
QPS3 QPS7 H10
QPS5 QPS6 H11
QPS6 QPS7 H12

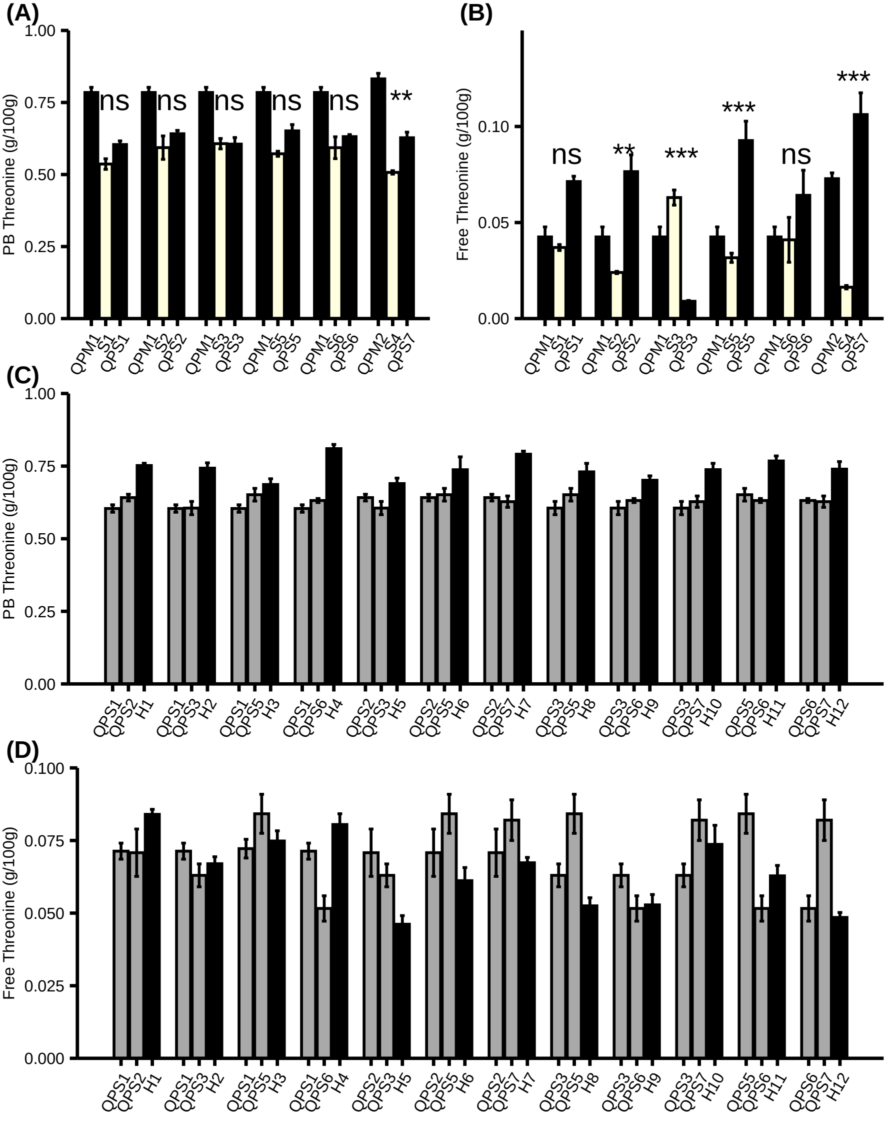
(A)
PB Threonine (g/100g)
1.00
0.75
0.50
0.25
0.00
ns
ns
ns
ns
ns
**
QPM1 S1 QPS1 QPM1 S2 QPS2 QPM1 S3 QPS3 QPM1 S5 QPS5 QPM1 S6 QPS6 QPM2 S4 QPS7
(B)
Free Threonine (g/100g)
0.10
0.05
0.00
ns
**
***
***
ns
***
QPM1 S1 QPS1 QPM1 S2 QPS2 QPM1 S3 QPS3 QPM1 S5 QPS5 QPM1 S6 QPS6 QPM2 S4 QPS7
(C)
PB Threonine (g/100g)
1.00
0.75
0.50
0.25
0.00
QPS1 QPS2 H1 QPS1 QPS3 H2 QPS1 QPS5 H3 QPS1 QPS6 H4 QPS2 QPS3 H5 QPS2 QPS5 H6 QPS2 QPS7 H7 QPS3 QPS5 H8 QPS3 QPS6 H9 QPS3 QPS7 H10 QPS5 QPS6 H11 QPS6 QPS7 H12
(D)
Free Threonine (g/100g)
0.100
0.075
0.050
0.025
0.000
QPS1 QPS2 H1 QPS1 QPS3 H2 QPS1 QPS5 H3 QPS1 QPS6 H4 QPS2 QPS3 H5 QPS2 QPS5 H6 QPS2 QPS7 H7 QPS3 QPS5 H8 QPS3 QPS6 H9 QPS3 QPS7 H10 QPS5 QPS6 H11 QPS6 QPS7 H12

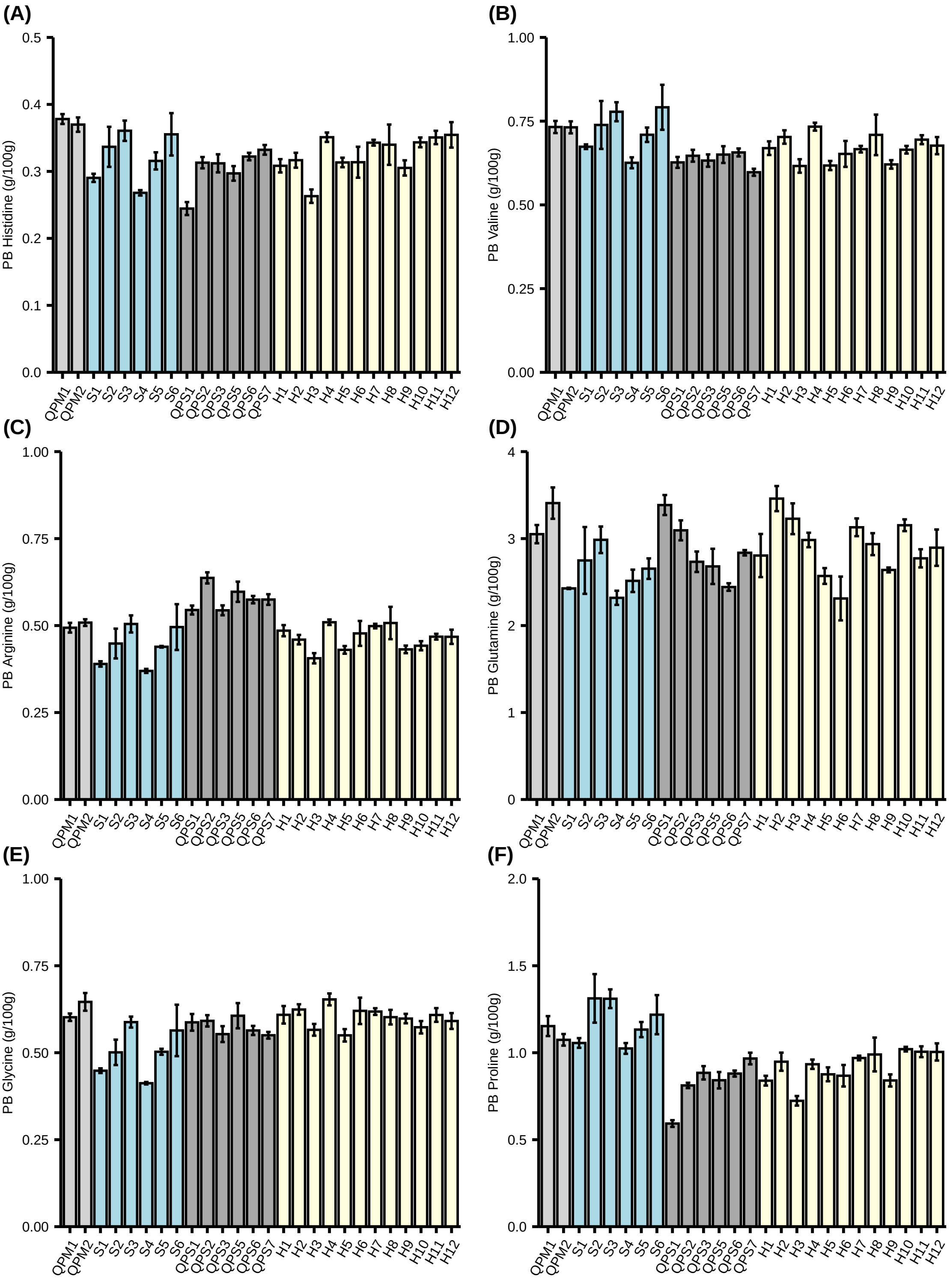

(A)
PB Histidine (g/100g)
(B)
PB Valine (g/100g)
(C)
PB Arginine (g/100g)
(D)
PB Glutamine (g/100g)
(E)
PB Glycine (g/100g)
(F)
PB Proline (g/100g)
QPM1 QPM2 S1 S2 S3 S4 S5 S6 QPS1 QPS2 QPS3 QPS5 QPS6 QPS7 H1 H2 H3 H4 H5 H6 H7 H8 H9 H10 H11 H12

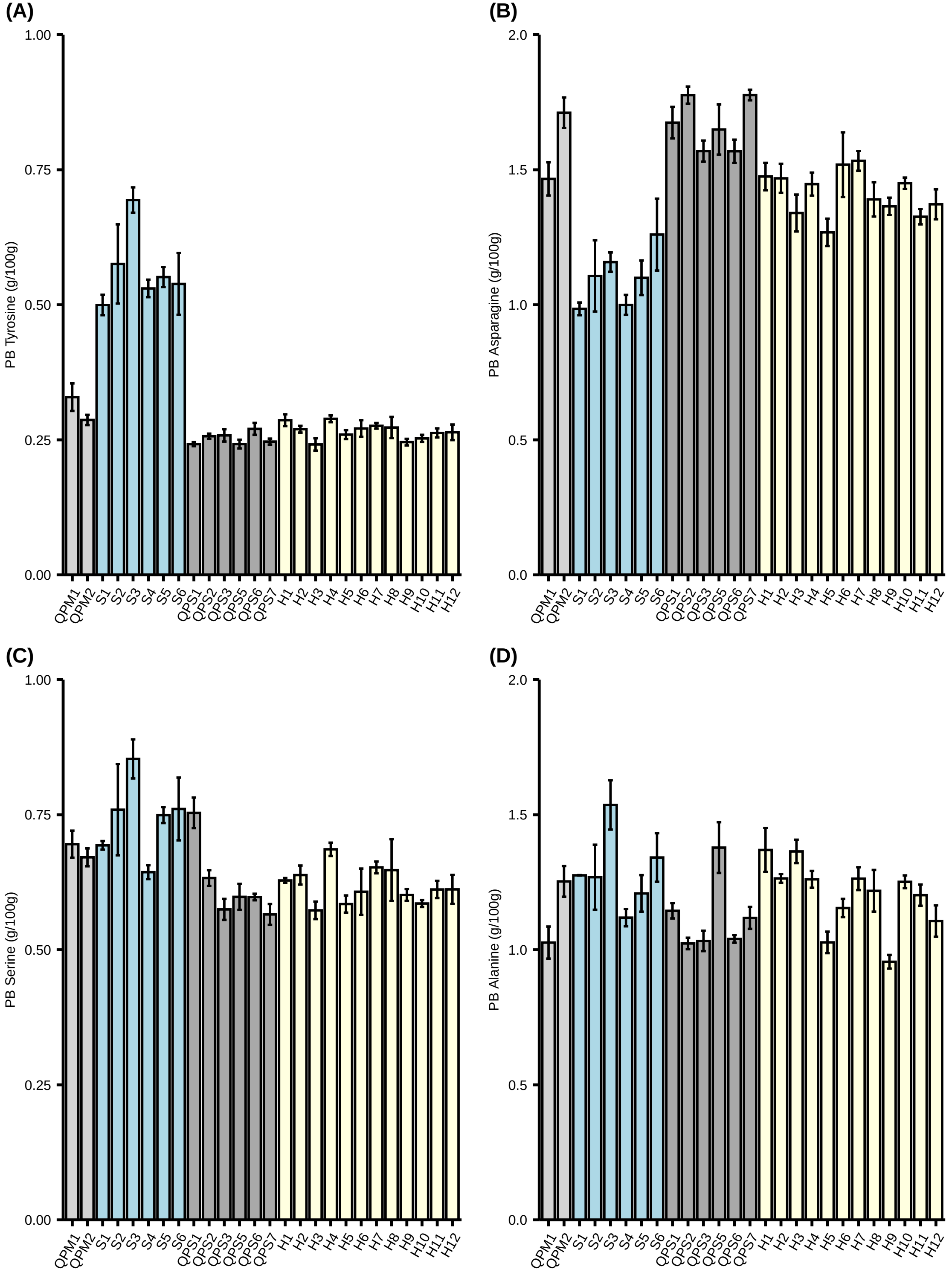
(A)
PB Tyrosine (g/100g)
0.00
0.25
0.50
0.75
1.00
QPM1
QPM2
S1
S2
S3
S4
S5
S6
QPS1
QPS2
QPS3
QPS5
QPS6
QPS7
H1
H2
H3
H4
H5
H6
H7
H8
H9
H10
H11
H12
(B)
PB Asparagine (g/100g)
0.0
0.5
1.0
1.5
2.0
(C)
PB Serine (g/100g)
0.00
0.25
0.50
0.75
1.00
(D)
PB Alanine (g/100g)
0.0
0.5
1.0
1.5
2.0

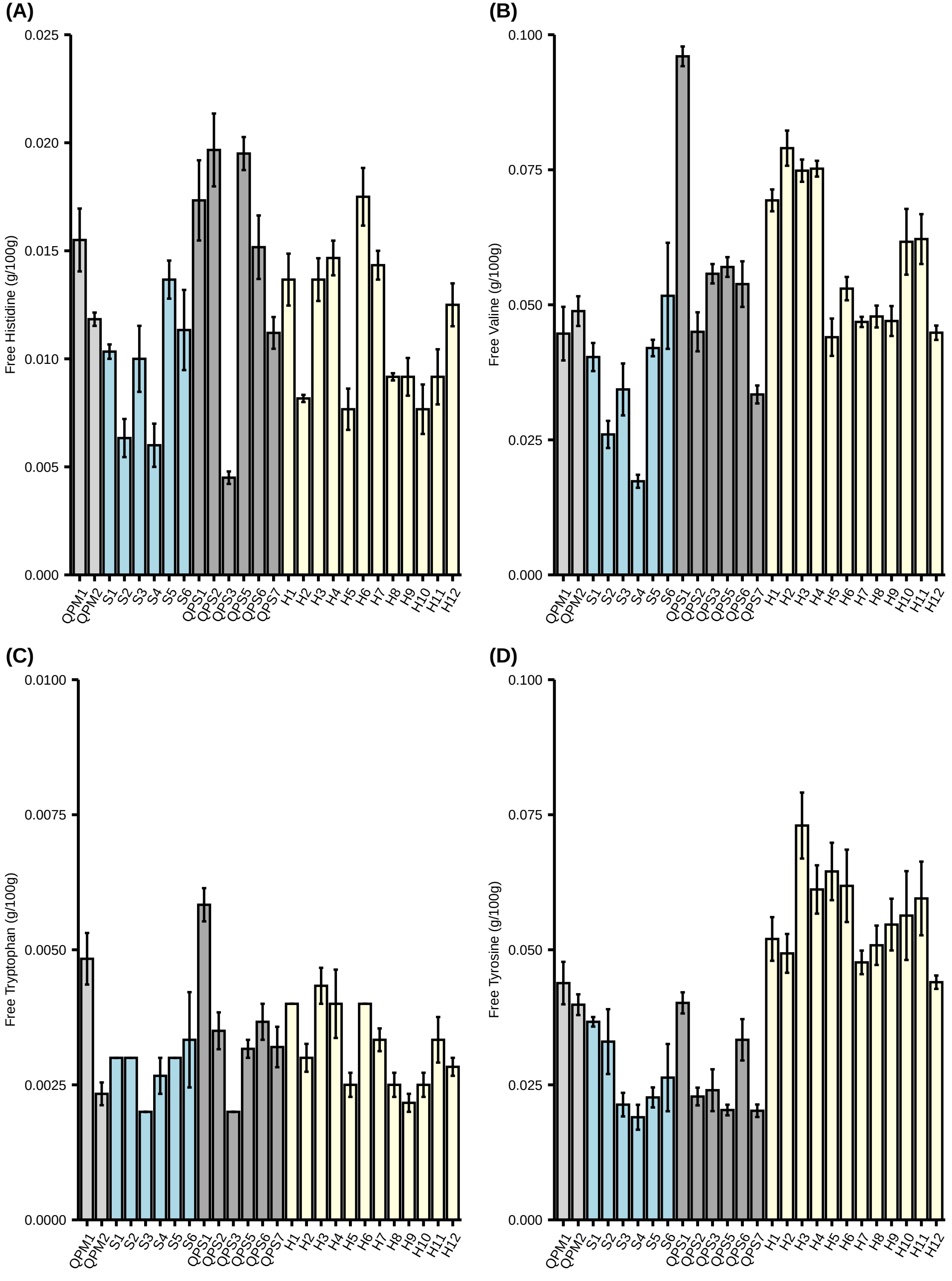

(A)
Free Histidine (g/100g)
0.000
0.005
0.010
0.015
0.020
0.025
QPM1 QPM2 S1 S2 S3 S4 S5 S6 QPS1 QPS2 QPS3 QPS5 QPS6 QPS7 H1 H2 H3 H4 H5 H6 H7 H8 H9 H10 H11 H12
(B)
Free Valine (g/100g)
0.000
0.025
0.050
0.075
0.100
QPM1 QPM2 S1 S2 S3 S4 S5 S6 QPS1 QPS2 QPS3 QPS5 QPS6 QPS7 H1 H2 H3 H4 H5 H6 H7 H8 H9 H10 H11 H12
(C)
Free Tryptophan (g/100g)
0.0000
0.0025
0.0050
0.0075
0.0100
QPM1 QPM2 S1 S2 S3 S4 S5 S6 QPS1 QPS2 QPS3 QPS5 QPS6 QPS7 H1 H2 H3 H4 H5 H6 H7 H8 H9 H10 H11 H12
(D)
Free Tyrosine (g/100g)
0.000
0.025
0.050
0.075
0.100
QPM1 QPM2 S1 S2 S3 S4 S5 S6 QPS1 QPS2 QPS3 QPS5 QPS6 QPS7 H1 H2 H3 H4 H5 H6 H7 H8 H9 H10 H11 H12

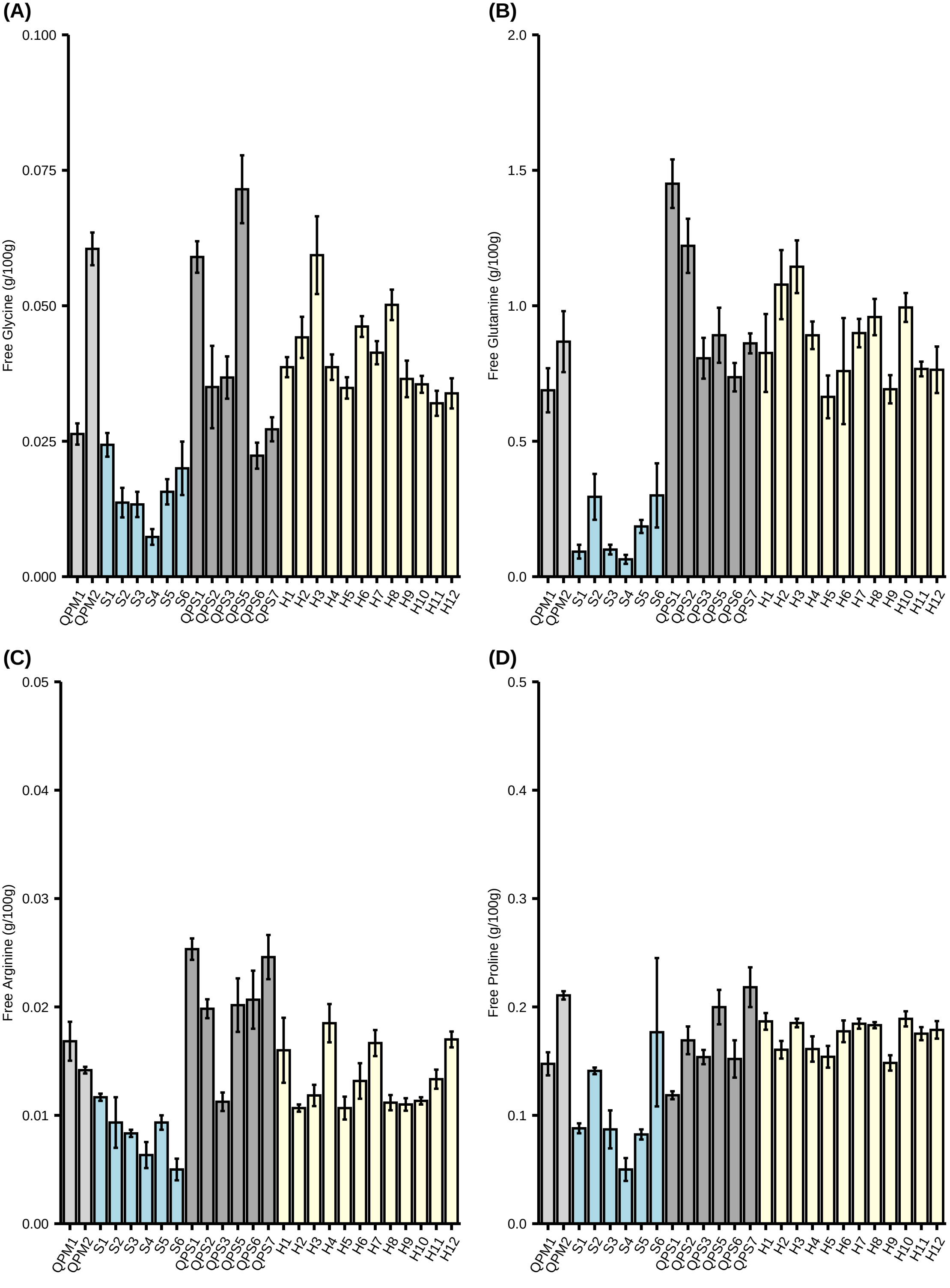
(A)
Free Glycine (g/100g)
0.000
0.025
0.050
0.075
0.100
QPM1 QPM2 S1 S2 S3 S4 S5 S6 QPS1 QPS2 QPS3 QPS5 QPS6 QPS7 H1 H2 H3 H4 H5 H6 H7 H8 H9 H10 H11 H12
(B)
Free Glutamine (g/100g)
0.0
0.5
1.0
1.5
2.0
QPM1 QPM2 S1 S2 S3 S4 S5 S6 QPS1 QPS2 QPS3 QPS5 QPS6 QPS7 H1 H2 H3 H4 H5 H6 H7 H8 H9 H10 H11 H12
(C)
Free Arginine (g/100g)
0.00
0.01
0.02
0.03
0.04
0.05
QPM1 QPM2 S1 S2 S3 S4 S5 S6 QPS1 QPS2 QPS3 QPS5 QPS6 QPS7 H1 H2 H3 H4 H5 H6 H7 H8 H9 H10 H11 H12
(D)
Free Proline (g/100g)
0.0
0.1
0.2
0.3
0.4
0.5
QPM1 QPM2 S1 S2 S3 S4 S5 S6 QPS1 QPS2 QPS3 QPS5 QPS6 QPS7 H1 H2 H3 H4 H5 H6 H7 H8 H9 H10 H11 H12

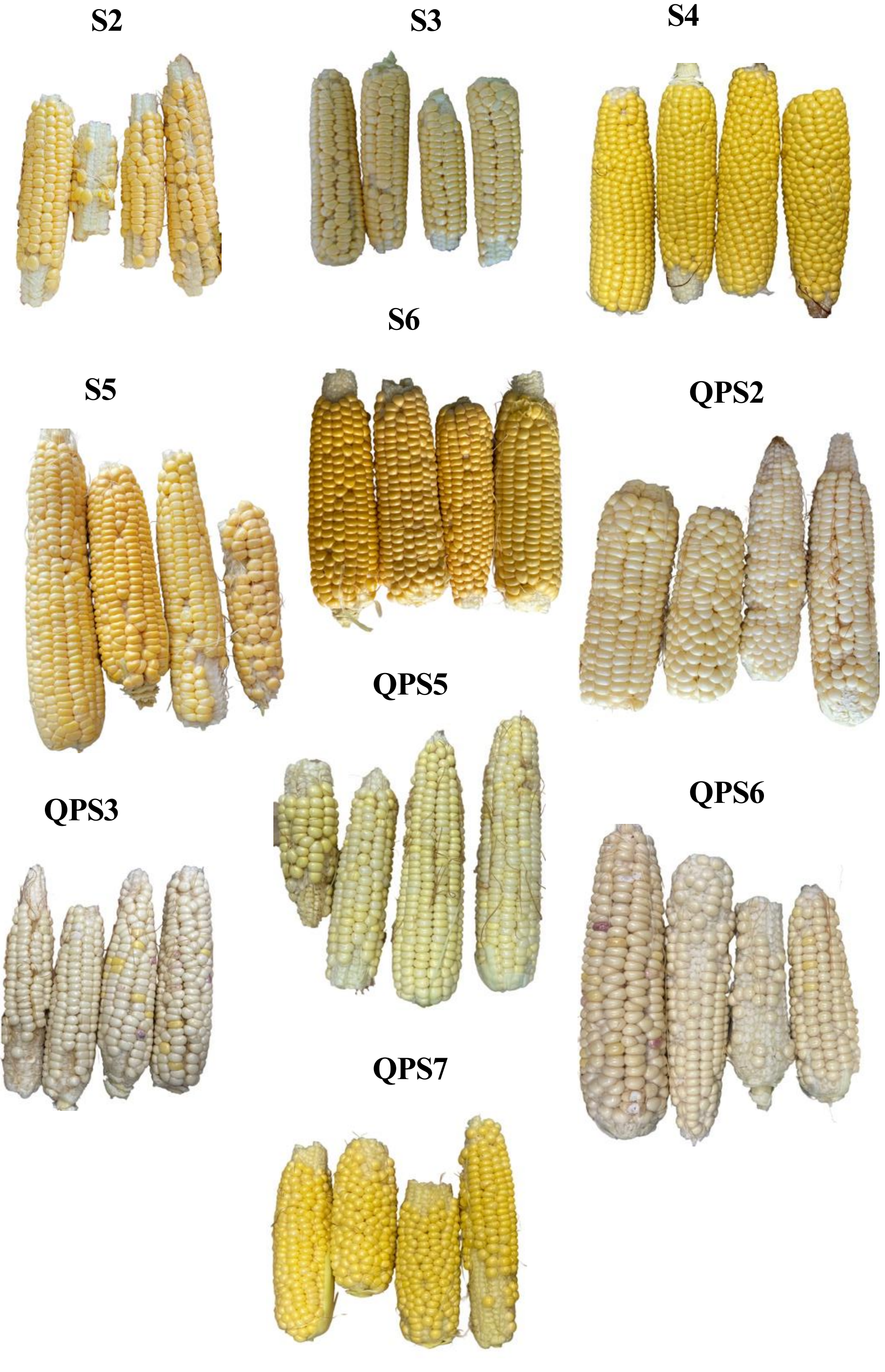
S2
S3
S4
S6
S5
QPS2
QPS5
QPS3
QPS6
QPS7

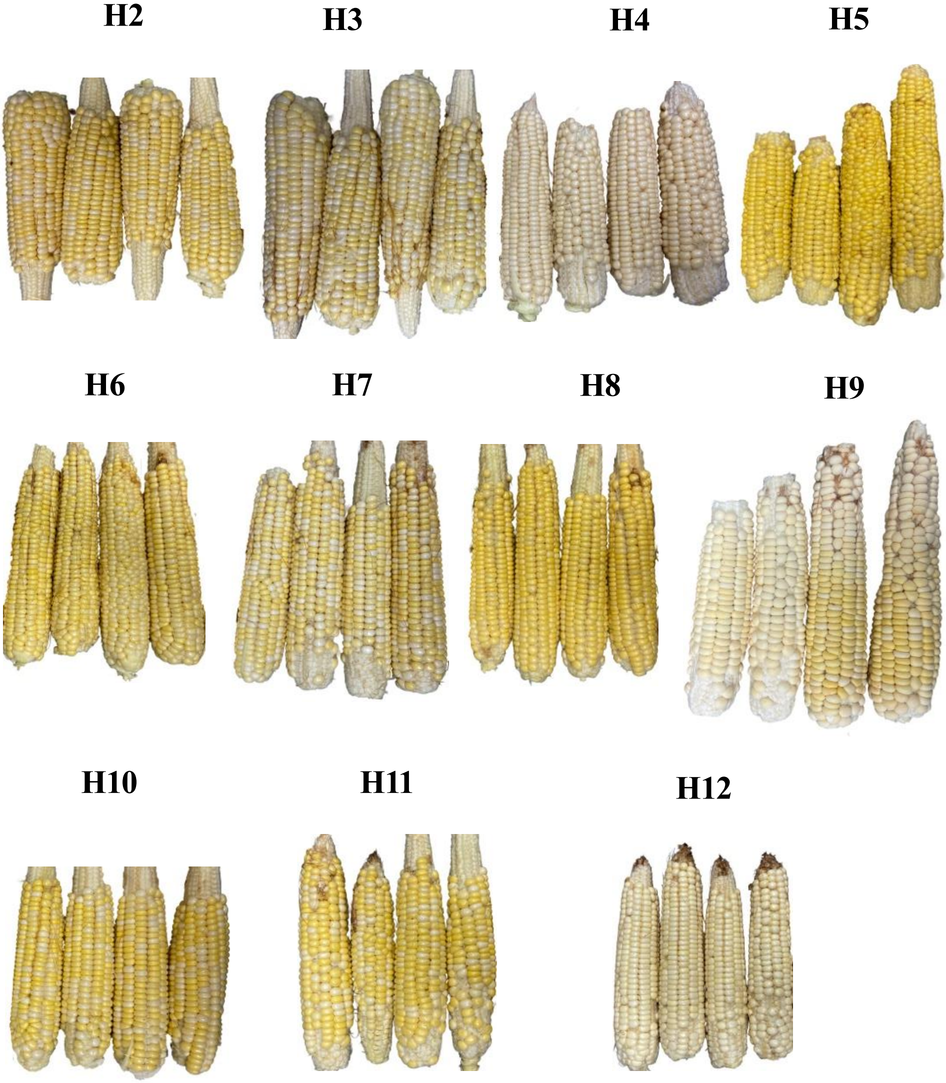
H2
H3
H4
H5
H6
H7
H8
H9
H10
H11
H12
