# Supplementary material for: Opaque-2 induced zein reduction and lysine increase suggest a route to quality protein sweet corn
Source: Front Plant Sci. 2026 May 14;17:1814115. doi: 10.3389/fpls.2026.1814115 (PMC13216212; doi:10.3389/fpls.2026.1814115)
Supplement: Supplementary Table 1 — Mean and sd values of protein-bound amino acids (g/100g dry weight) in parental sweet corn, QPS inbreds and hybrids. [file Table1.pdf]

Ref no.	Ala	Arg	Asx	Glx	Gly	His	Ile	Leu	Lys	Met	Phe	Pro	Ser	Thr	Tyr	Val
**QPM1**	1.027	0.494	1.466	3.051	0.602	0.378	0.571	1.293	0.666	0.345	0.613	1.153	0.695	0.785	0.329	0.733
**QPM2**	1.253	0.508	1.711	3.408	0.646	0.370	0.538	1.058	0.784	0.306	0.552	1.074	0.671	0.832	0.287	0.731
**S1**	1.276	0.389	0.985	2.428	0.449	0.290	0.562	1.485	0.522	0.243	0.616	1.056	0.693	0.536	0.500	0.674
**S2**	1.269	0.449	1.107	2.749	0.501	0.337	0.644	1.700	0.531	0.287	0.676	1.312	0.759	0.593	0.576	0.739
**S3**	1.536	0.505	1.158	2.986	0.588	0.361	0.694	1.870	0.517	0.378	0.699	1.310	0.853	0.607	0.694	0.778
**S4**	1.119	0.370	1.000	2.319	0.412	0.268	0.548	1.585	0.405	0.273	0.577	1.024	0.644	0.508	0.530	0.625
**S5**	1.209	0.439	1.100	2.514	0.503	0.316	0.625	1.661	0.506	0.272	0.664	1.133	0.749	0.572	0.551	0.709
**S6**	1.341	0.496	1.260	2.654	0.564	0.355	0.638	1.535	0.624	0.284	0.638	1.219	0.760	0.593	0.539	0.791
**QPS1**	1.144	0.544	1.674	3.386	0.588	0.245	0.365	0.751	0.627	0.333	0.422	0.593	0.753	0.604	0.242	0.626
**QPS2**	1.023	0.637	1.776	3.095	0.592	0.313	0.397	0.855	0.663	0.312	0.427	0.812	0.633	0.642	0.257	0.647
**QPS3**	1.033	0.544	1.569	2.734	0.554	0.312	0.370	0.841	0.549	0.225	0.397	0.884	0.575	0.605	0.258	0.632
**QPS5**	1.378	0.597	1.741	2.680	0.606	0.297	0.394	0.820	0.717	0.242	0.431	0.842	0.598	0.651	0.242	0.650
**QPS6**	1.040	0.575	1.569	2.443	0.564	0.322	0.396	0.883	0.679	0.250	0.415	0.880	0.598	0.631	0.270	0.657
**QPS7**	1.118	0.575	1.831	2.837	0.550	0.332	0.369	0.831	0.629	0.205	0.396	0.967	0.565	0.627	0.247	0.597
**H1**	1.370	0.485	1.475	2.805	0.609	0.308	0.449	0.845	0.692	0.307	0.472	0.840	0.628	0.752	0.286	0.669
**H2**	1.264	0.459	1.468	3.460	0.624	0.317	0.458	0.884	0.651	0.288	0.480	0.948	0.638	0.743	0.270	0.702
**H3**	1.364	0.406	1.340	3.228	0.566	0.263	0.404	0.754	0.635	0.260	0.443	0.724	0.573	0.687	0.242	0.616
**H4**	1.261	0.509	1.447	2.984	0.653	0.351	0.490	0.944	0.722	0.315	0.513	0.934	0.686	0.810	0.289	0.734
**H5**	1.027	0.430	1.268	2.570	0.550	0.314	0.426	0.844	0.614	0.249	0.447	0.876	0.585	0.690	0.260	0.617
**H6**	1.155	0.477	1.629	2.641	0.620	0.314	0.474	0.880	0.682	0.305	0.492	0.868	0.607	0.737	0.271	0.652
**H7**	1.263	0.499	1.533	3.130	0.618	0.343	0.477	0.922	0.702	0.321	0.495	0.970	0.652	0.791	0.276	0.666
**H8**	1.218	0.507	1.530	3.272	0.673	0.340	0.492	0.953	0.735	0.295	0.530	0.990	0.648	0.801	0.273	0.709
**H9**	0.956	0.432	1.365	2.639	0.598	0.305	0.429	0.832	0.606	0.297	0.450	0.841	0.601	0.701	0.246	0.621
**H10**	1.252	0.442	1.450	3.153	0.573	0.343	0.438	0.873	0.631	0.262	0.458	1.021	0.586	0.738	0.253	0.665
**H11**	1.202	0.468	1.326	2.773	0.609	0.351	0.475	0.932	0.659	0.294	0.486	1.006	0.612	0.768	0.263	0.695
**H12**	1.106	0.468	1.372	2.896	0.591	0.355	0.482	0.942	0.684	0.279	0.497	1.005	0.612	0.740	0.264	0.677

Mean difference percentage

	Ala	Arg	Asx	Glx	Gly	His	Ile	Leu	Lys	Met	Phe	Pro	Ser	Thr	Tyr	Val
S1-QPS1	10.3	-39.8	-70	-39	-30.9	15.77	34.9	49.4	-20.3	-36.9	31.5	43.9	-8.6	-12.6	51.6	7
S2-QPS2	19.35	-42	-60.5	-13	-18	7.058	38.4	49.7	-24.8	-8.58	36.9	38.1	16.7	-8.12	55.5	12.4
S3-QPS3	32.76	-7.73	-35.5	8.45	5.83	13.53	46.7	55.1	-6.23	40.61	43.2	32.5	32.6	0.27	62.8	18.7
S5-QPS5	-14	-36	-58.2	-6.6	-20.6	5.91	37	50.6	-41.8	11.19	35.1	25.7	20.2	-13.9	56.1	8.33
S6-QPS6	22.44	-15.9	-24.5	7.95	0.06	9.414	37.9	42.5	-8.72	12.06	34.9	27.8	21.4	-6.45	49.8	17
S4-QPS7	0.082	-55.5	-83.2	-22	-33.5	-23.8	32.6	47.6	-55.3	25	31.3	5.63	12.2	-23.6	53.5	4.47

Ref no.	Ala	Arg	Asn	Asp	Gln	Glu	Gly	His	Ile	Leu	Lys	Met	Phe	Pro	Ser	Trp	Thr	Tyr	Val	Cys
QPM1	0.161	0.017	0.109	0.238	0.688	0.340	0.026	0.015	0.014	0.022	0.057	0.019	0.011	0.147	0.102	0.005	0.042	0.044	0.045	0.001
QPM2	0.393	0.014	0.188	0.236	0.868	0.648	0.060	0.012	0.014	0.025	0.063	0.031	0.008	0.210	0.103	0.002	0.073	0.040	0.049	0.001
S1	0.258	0.012	0.034	0.095	0.092	0.252	0.025	0.010	0.013	0.020	0.063	0.011	0.018	0.088	0.079	0.003	0.037	0.037	0.040	0.001
S2	0.151	0.009	0.030	0.086	0.295	0.189	0.014	0.006	0.012	0.018	0.047	0.016	0.007	0.141	0.055	0.003	0.024	0.033	0.026	0.001
S3	0.266	0.009	0.036	0.097	0.100	0.179	0.013	0.010	0.010	0.015	0.042	0.020	0.008	0.087	0.073	0.002	0.033	0.021	0.034	0.001
S4	0.110	0.006	0.036	0.113	0.064	0.079	0.007	0.006	0.007	0.009	0.034	0.004	0.007	0.050	0.044	0.003	0.016	0.019	0.018	0.001
S5	0.138	0.010	0.059	0.098	0.185	0.124	0.015	0.014	0.011	0.015	0.051	0.014	0.008	0.082	0.071	0.003	0.032	0.023	0.042	0.001
S6	0.242	0.005	0.051	0.153	0.300	0.215	0.020	0.011	0.012	0.020	0.038	0.021	0.012	0.177	0.075	0.003	0.041	0.026	0.052	0.001
QPS1	0.451	0.025	0.205	0.000	1.451	0.000	0.059	0.017	0.022	0.047	0.035	0.109	0.044	0.119	0.281	0.006	0.071	0.040	0.096	0.006
QPS2	0.303	0.020	0.095	0.000	1.221	0.000	0.035	0.020	0.018	0.037	0.030	0.070	0.011	0.169	0.131	0.003	0.076	0.023	0.045	0.003
QPS3	0.349	0.011	0.062	0.000	0.806	0.000	0.037	0.005	0.011	0.023	0.022	0.017	0.009	0.154	0.103	0.002	0.063	0.024	0.056	0.002
QPS5	0.543	0.020	0.096	0.000	0.891	0.059	0.072	0.019	0.014	0.027	0.055	0.024	0.019	0.200	0.101	0.003	0.093	0.020	0.057	0.004
QPS6	0.347	0.021	0.117	0.081	0.736	0.165	0.022	0.015	0.021	0.024	0.069	0.033	0.011	0.152	0.104	0.004	0.064	0.033	0.054	0.002
QPS7	0.383	0.025	0.187	0.000	0.861	0.000	0.027	0.011	0.010	0.020	0.052	0.017	0.010	0.218	0.093	0.003	0.106	0.020	0.033	0.002
H1	0.548	0.016	0.111	0.285	0.826	0.422	0.039	0.014	0.020	0.035	0.036	0.049	0.016	0.187	0.122	0.004	0.084	0.052	0.069	0.002
H2	0.468	0.011	0.105	0.273	1.078	0.634	0.044	0.008	0.015	0.030	0.024	0.028	0.017	0.161	0.123	0.003	0.067	0.049	0.079	0.001
H3	0.588	0.012	0.090	0.259	1.144	0.597	0.059	0.014	0.018	0.035	0.031	0.029	0.023	0.185	0.123	0.004	0.075	0.073	0.075	0.002
H4	0.462	0.018	0.103	0.249	0.891	0.448	0.038	0.015	0.020	0.033	0.041	0.036	0.018	0.161	0.148	0.004	0.081	0.061	0.080	0.002
H5	0.316	0.011	0.059	0.276	0.664	0.469	0.035	0.008	0.014	0.024	0.029	0.022	0.010	0.154	0.111	0.002	0.046	0.065	0.044	0.001
H6	0.390	0.013	0.121	0.372	0.759	0.359	0.046	0.018	0.020	0.033	0.030	0.030	0.018	0.177	0.102	0.004	0.061	0.062	0.053	0.001
H7	0.444	0.017	0.105	0.314	0.899	0.510	0.041	0.014	0.016	0.030	0.035	0.046	0.012	0.185	0.117	0.003	0.067	0.048	0.047	0.001
H8	0.369	0.011	0.062	0.321	0.958	0.465	0.050	0.009	0.012	0.026	0.028	0.018	0.018	0.183	0.094	0.002	0.052	0.051	0.048	0.001
H9	0.269	0.011	0.063	0.310	0.692	0.407	0.037	0.009	0.015	0.027	0.022	0.034	0.010	0.148	0.120	0.002	0.053	0.055	0.047	0.001
H10	0.494	0.011	0.133	0.312	0.994	0.527	0.035	0.008	0.016	0.024	0.043	0.030	0.013	0.189	0.108	0.002	0.074	0.056	0.062	0.002
H11	0.416	0.013	0.088	0.235	0.767	0.411	0.032	0.009	0.018	0.026	0.043	0.032	0.011	0.175	0.107	0.003	0.063	0.059	0.062	0.001
H12	0.352	0.017	0.085	0.239	0.764	0.457	0.034	0.013	0.014	0.023	0.048	0.030	0.009	0.179	0.102	0.003	0.049	0.044	0.045	0.001

Mean differene Percentage

	Ala	Arg	Asn	Asp	Gln	Glu	Gly	His	Ile	Leu	Lys	Met	Phe	Pro	Ser	Trp	Thr	Tyr	Val	Cys
S1-QPS1	-75.01	-119.83	-503.45	100.00	-1475.10	100.00	-141.71	-67.61	-66.18	-137.57	45.07	-885.36	-140.71	-34.45	-255.88	-94.85	-92.16	-9.12	-139.00	-411.78
S2-QPS2	-100.49	-110.09	-220.40	100.00	-314.12	100.00	-152.72	-217.12	-49.32	-105.85	36.07	-330.59	-56.44	-20.08	-139.93	-19.34	-217.72	30.48	-73.90	-198.74
S3-QPS3	-30.94	-34.09	-72.41	100.00	-706.22	100.00	-185.30	53.11	-14.67	-57.61	48.40	15.61	-10.04	-76.86	-41.78	-3.38	-89.93	-11.33	-63.08	-22.82
S5-QPS5	-293.46	-112.48	-64.41	100.00	-380.87	52.28	-366.71	-42.96	-32.76	-86.47	-8.88	-73.70	-130.27	-143.43	-42.14	-15.73	-193.15	9.91	-35.79	-317.30
S6-QPS6	-43.71	-324.49	-127.46	46.84	-145.50	23.24	-12.16	-33.99	-77.36	-18.69	-79.88	-62.62	12.87	14.01	-39.45	-7.82	-57.42	-26.84	-4.47	-44.28
S4-QPS7	-247.09	-282.20	-420.69	100.00	-1238.47	100.00	-265.97	-79.46	-33.49	-131.34	-53.63	-306.42	-44.49	-336.80	-110.80	-23.25	-548.46	-3.85	-89.51	-153.33

PBAA/FAA Ratios

PBAA/FAA	Ala	Arg	Asx	Gln	His	Ile	Leu	Lys	Met	Phe	Pro	Ser	Thr	Tyr	Val
**S1**	5	34	29	5	28	43	76	8	22	34	12	9	14	14	17
**QPS1**	3	22	8	0	14	17	16	18	3	10	5	3	8	6	7
**S2**	8	48	37	2	54	55	94	11	18	94	9	14	25	17	29
**QPS2**	3	32	19	0	16	23	23	22	4	38	5	5	8	11	14
**S3**	6	59	32	6	37	72	128	12	19	85	15	12	18	32	23
**QPS3**	3	48	25	1	68	33	37	26	13	44	6	6	10	11	11
**S5**	9	46	19	3	23	58	115	10	20	80	14	11	18	24	17
**QPS5**	3	30	18	1	15	27	30	13	10	22	4	6	7	12	11
**S6**	6	101	24	2	31	54	75	16	14	52	7	10	15	20	15
**QPS6**	3	28	13	1	21	19	36	10	7	39	6	6	10	8	12
**S4**	10	58	28	6	42	76	181	12	65	85	20	15	31	27	36
**QPS7**	3	23	10	1	29	39	41	12	12	40	4	6	6	12	18

Ear Weight Comparison among QPS Hybrids (*p* -values)

Hybrids	H1	H2	H3	H4	H5	H6	H7	H8	H9	H10	H11
H1											
H2	1.000										
H3	0.805	0.164									
H4	0.914	0.297	1.000								
H5	1.000	1.000	0.284	0.463							
H6	0.975	0.493	1.000	1.000	0.679						
H7	0.994	0.672	0.999	1.000	0.835	1.000					
H8	0.278	0.011	0.996	0.972	0.025	0.883	0.747				
H9	0.984	0.555	1.000	1.000	0.738	1.000	1.000	0.842			
H10	0.055	0.001	0.748	0.549	0.002	0.343	0.210	0.999	0.293		
H11	0.035	0.000	0.615	0.414	0.001	0.237	0.136	0.994	0.198	1.000	
H12	0.249	0.009	0.994	0.959	0.020	0.852	0.703	1.000	0.806	1.000	0.997

Ear Length Comparison among QPS Hybrids (*p* -values)

Hybrids	H1	H2	H3	H4	H5	H6	H7	H8	H9	H10	H11
H1											
H2	0.807										
H3	1.000	0.948									
H4	1.000	0.925	1.000								
H5	0.945	0.009	0.329	0.380							
H6	0.875	0.004	0.204	0.241	1.000						
H7	0.446	0.000	0.025	0.032	0.993	0.999					
H8	0.998	0.050	0.718	0.770	1.000	0.999	0.859				
H9	0.768	0.002	0.117	0.142	1.000	1.000	1.000	0.993			
H10	0.726	1.000	0.895	0.859	0.005	0.002	0.000	0.032	0.001		
H11	1.000	0.329	0.993	0.997	0.948	0.859	0.329	0.999	0.718	0.241	
H12	1.000	0.204	0.966	0.979	0.988	0.948	0.489	1.000	0.859	0.142	1.000

Protein-bound amino ac

Ratios	Ala	Arg	Asx	Glx	Gly	His	Ile	Leu	Lys	Met	Phe	Pro	Ser	Thr	Tyr	Val
**QPS1/S1**	0.90	1.40	1.70	1.39	1.31	0.84	0.65	0.51	1.20	1.37	0.69	0.56	1.09	1.13	0.48	0.93
**QPS2/S2**	0.81	1.42	1.60	1.13	1.18	0.93	0.62	0.50	1.25	1.09	0.63	0.62	0.83	1.08	0.45	0.88
**QPS3/S3**	0.67	1.08	1.35	0.92	0.94	0.86	0.53	0.45	1.06	0.59	0.57	0.68	0.67	1.00	0.37	0.81
**QPS5/S5**	1.14	1.36	1.58	1.07	1.21	0.94	0.63	0.49	1.42	0.89	0.65	0.74	0.80	1.14	0.44	0.92
**QPS6/S6**	0.78	1.16	1.24	0.92	1.00	0.91	0.62	0.58	1.09	0.88	0.65	0.72	0.79	1.06	0.50	0.83
**QPS7/S4**	1.00	1.55	1.83	1.22	1.33	1.24	0.67	0.52	1.55	0.75	0.69	0.94	0.88	1.24	0.47	0.96

Free amino acid

Ratios	Ala	Arg	Asn	Asp	Gln	Glu	Gly	His	Ile	Leu	Lys	Met	Phe	Pro	Ser	Trp	Thr	Tyr	Val	Cys
**QPS1/S1**	1.75	2.20	6.03	0.00	15.75	0.00	2.42	1.68	1.66	2.38	0.55	9.85	2.41	1.34	3.56	1.95	1.92	1.09	2.39	5.12
**QPS2/S2**	2.00	2.10	3.20	0.00	4.14	0.00	2.53	3.17	1.49	2.06	0.64	4.31	1.56	1.20	2.40	1.19	3.18	0.70	1.74	2.99
**QPS3/S3**	1.31	1.34	1.72	0.00	8.06	0.00	2.85	0.47	1.15	1.58	0.52	0.84	1.10	1.77	1.42	1.03	1.90	1.11	1.63	1.23
**QPS5/S5**	3.93	2.12	1.64	0.00	4.81	0.48	4.67	1.43	1.33	1.86	1.09	1.74	2.30	2.43	1.42	1.16	2.93	0.90	1.36	4.17
**QPS6/S6**	1.44	4.24	2.27	0.53	2.46	0.77	1.12	1.34	1.77	1.19	1.80	1.63	0.87	0.86	1.39	1.08	1.57	1.27	1.04	1.44
**QPS7/S4**	3.47	3.82	5.21	0.00	13.38	0.00	3.66	1.79	1.33	2.31	1.54	4.06	1.44	4.37	2.11	1.23	6.48	1.04	1.90	2.53
